# G proteins of the G_12_ family expressed by POMC neurons regulate key metabolic functions

**DOI:** 10.1126/sciadv.adu1670

**Published:** 2025-07-11

**Authors:** Dhanush Haspula, Zhenzhong Cui, Srinivas Pittala, Yinghong Cui, Huiyan Lu, Yan Xiong, Jian Jin, Oksana Gavrilova, Eunsang Hwang, Jason Ajwani, Bryan Portillo, Kevin W. Williams, Asuka Inoue, Jürgen Wess

**Affiliations:** ^1^ Molecular Signaling Section, Laboratory of Bioorganic Chemistry, National Institute of Diabetes and Digestive and Kidney Diseases, Bethesda, MD 20892, USA.; ^2^ Mouse Metabolism Core, National Institute of Diabetes and Digestive and Kidney Diseases, Bethesda, MD 20892, USA.; ^3^ Mouse Transgenic Core Facility, National Institute of Diabetes and Digestive and Kidney Diseases, Bethesda, MD 20892, USA.; ^4^ Mount Sinai Center for Therapeutics Discovery, Departments of Pharmacological Sciences and Oncological Sciences, Tisch Cancer Institute, Icahn School of Medicine at Mount Sinai, New York, NY 10029, USA.; ^5^ Center for Hypothalamic Research, Department of Internal Medicine, Peter O’Donnell Jr. Brain Institute, The University of Texas Southwestern Medical Center, Dallas, TX 75390, USA.; ^6^ Graduate School of Pharmaceutical Sciences, Tohoku University, Sendai, Miyagi 980-8578, Japan.; ^7^ Graduate School of Pharmaceutical Sciences, Kyoto University, Kyoto 606-8501, Japan.

## Abstract

Proopiomelanocortin (POMC) neurons play a key role in maintaining glucose and energy homeostasis. POMC neurons express many heterotrimeric guanine nucleotide–binding protein (G protein)–coupled receptors that are linked to different functional classes of G proteins. The potential role of G_12/13_ in regulating the function of central POMC neurons remains unknown. To address this question, we used a chemogenetic approach to selectively stimulate G_12/13_ signaling in POMC neurons. We found that receptor-mediated activation of G_12/13_ signaling in POMC neurons caused notable improvements in glucose homeostasis in lean and obese mice. Stimulation of G_12/13_ signaling in POMC neurons also enhanced the physiological actions of leptin. Studies with G_12/13_ knockout mice showed that G_12/13_ signaling in POMC neurons mediated the beneficial metabolic effects of lorcaserin, an appetite-suppressant drug that selectively activates serotonin 5-HT_2C_ receptors. These findings indicate that G_12/13_-coupled receptors expressed by POMC neurons represent potential targets for advanced classes of antidiabetic and appetite-suppressant drugs.

## Teaser

Activation of G proteins of the G_12_ family expressed by POMC neurons causes marked improvements in glucose homeostasis.

## INTRODUCTION

Heterotrimeric guanine nucleotide–binding protein (G protein)–coupled receptors (GPCRs) represent by far the largest class of plasma membrane receptors and are the target of about one-third of all drugs in current clinical use (*1*). GPCRs modulate virtually all major functions of the central nervous system (*2*, *3*), including the central regulation of energy and glucose homeostasis (*4*–*8*).

Distinct neuronal populations located within the arcuate nucleus of the hypothalamus (ARC) play preeminent roles in regulating food intake, energy expenditure, and glucose homeostasis (*9*–*11*). Numerous studies have shown that two major groups of ARC neurons are of particular importance in the control of these metabolic functions (*9*–*11*). One of these neuronal populations stores and releases agouti-related protein (AgRP), neuropeptide Y (NPY), and γ-aminobutyric acid (AgRP neurons). Activation of AgRP neurons by various factors including hormonal stimuli promotes food intake and decreased energy expenditure (*9*–*11*). The second major class of ARC neurons regulating glucose and energy homeostasis is referred to as proopiomelanocortin (POMC) neurons. POMC neurons are activated by certain nutrients or hormones (e.g., leptin or insulin), resulting in the release of α-melanocyte-stimulating hormone (α-MSH) in multiple brain areas (*9*–*11*). α-MSH then binds to and activates neurons expressing melanocortin-4 receptors (besides melanocortin-3 receptors), resulting in reduced food intake and increased energy expenditure (*5*).

Like essentially all other cell types, POMC neurons express dozens of distinct GPCRs (*12*). GPCRs are activated by the binding of specific extracellular ligands, including small molecules (e.g., neurotransmitters), regulatory peptides, or proteins (*1*). Following the binding of agonist ligands, GPCRs can interact with and activate one or more functional classes of G proteins, the G_s_, G_i_, G_q_, and G_12/13_ families (*13*). Several studies have explored the functional outcomes of activating G_s_-, G_i_-, or G_q_-coupled GPCRs in distinct neuronal populations of the ARC [e.g., (*14*–*16*)].

Although G proteins of the G_12_ family (G_12_ and G_13_) are widely expressed throughout the brain (*17*), the physiological or pathophysiological roles of neuronal G_12/13_ signaling remain largely unknown. Studies in this area have been hampered by several factors. First, G_12/13_-specific pharmacological inhibitors are not available at present. Second, GPCRs that exclusively couple to G_12/13_ have not been identified so far. Third, the α subunits of G_12_ and G_13_ (Gα_12_ and Gα_13_, respectively) share a high degree of sequence homogony and have similar functional properties (*18*), a fact that has greatly complicated the interpretation of gene knockout (KO) or knockdown studies.

According to the HypoMap single-cell gene expression atlas of the murine hypothalamus (*19*), both *Gna12* and *Gna13* (the genes encoding Gα_12_ and Gα_13_, respectively) are expressed by both neuronal and nonneuronal cells in the ARC. Moreover, transcriptomic analyses revealed that *Gna12* and *Gna13* are expressed at moderate levels by both AgRP and POMC neurons of adult mice (*12*).

Published transcriptomics data (*12*) also indicate that mouse ARC POMC neurons express relatively high levels of several receptors that can couple to G_12/13_, in addition to other functional classes of G proteins. These receptors include, for example, the cannabinoid receptor 1, the α_2A_-adrenoceptor, the 5-HT_2C_ receptor, and the hypocretin receptor 2 (gene names: *Cnr1*, *Adra2a*, *Htr2c*, and *Hcrtr2*, respectively) (*20*–*23*).

On the basis of these findings, the present study was designed to elucidate the potential metabolic roles of G_12_-type G proteins expressed by POMC neurons in regulating glucose and energy homeostasis. Detailed metabolic studies with several newly generated mouse models resulted in a number of interesting findings. Chemogenetic activation of G_12/13_ signaling in POMC neurons led to pronounced improvements in glucose homeostasis because of various mechanisms including changes in autonomic outflow. We also demonstrated that the beneficial metabolic effects of lorcaserin (Lorca), an appetite-suppressant drug, depend on the activation of G_12_-type G proteins in POMC neurons. These findings provide a rational basis for the development of previously unidentified classes of drugs aimed at enhancing G_12/13_ signaling in POMC neurons for the treatment of various metabolic disorders including obesity and type 2 diabetes.

## RESULTS

### Acute activation of G12D in POMC neurons causes a delayed hypophagic effect

Initially, we generated a mouse line that expressed a G_12/13_-coupled designer GPCR (*22*, *24*, *25*) selectively in POMC neurons. This designer GPCR, generally referred to as G12 DREADD (designer receptor exclusively activated by a designer drug; short: G12D), can be selectively activated by certain small molecules such as clozapine-*N*-oxide (CNO) or deschloroclozapine (DCZ) (*26*–*28*).

**Fig. 1.**
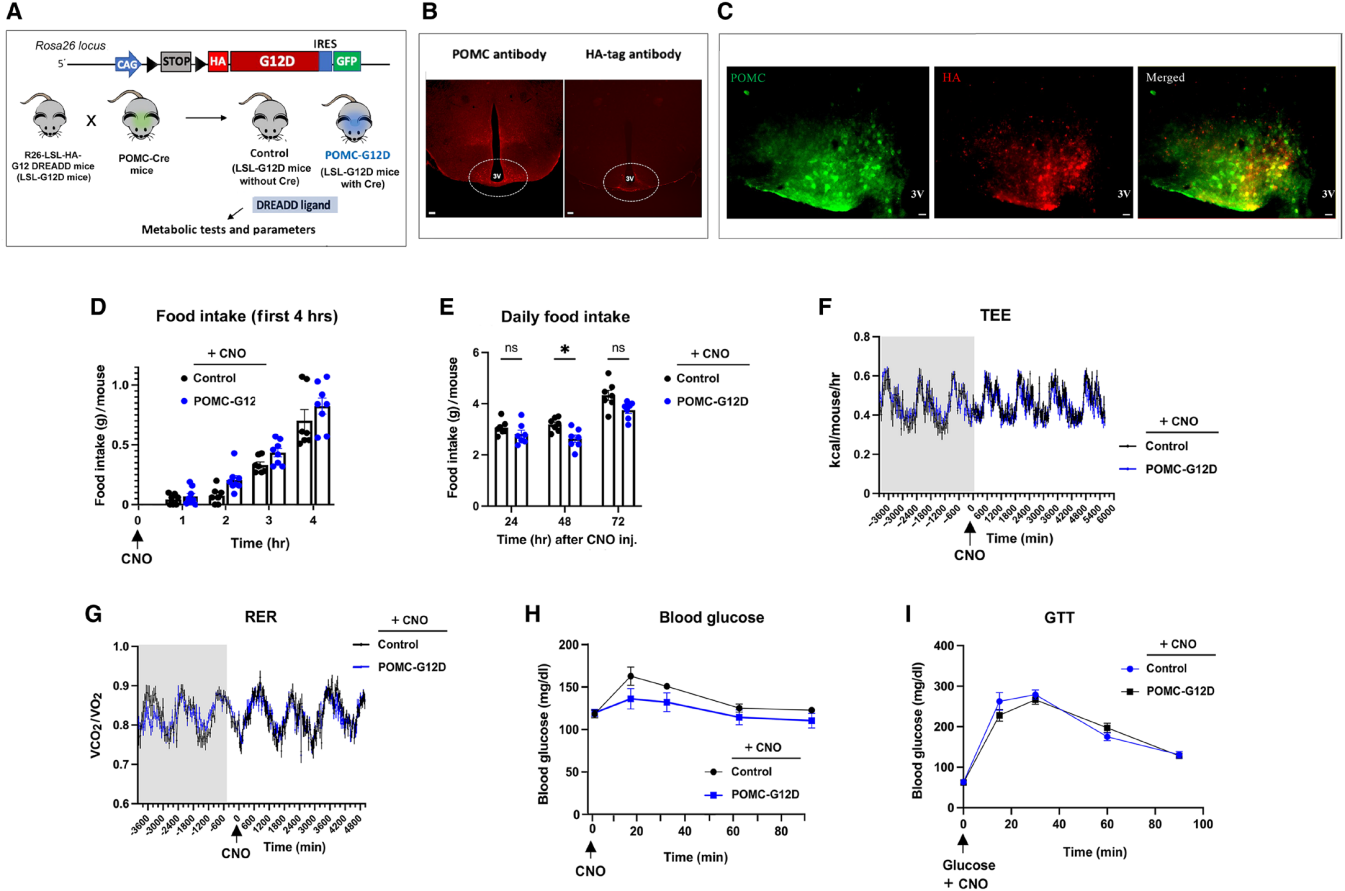
Generation and initial metabolic analysis of mice expressing the G12D designer receptor in POMC neurons. All experiments were carried out with male mice maintained on regular chow. (**A**) Scheme summarizing the strategy used to generate mice expressing G12D selectively in POMC neurons (POMC-G12D mice; see Materials and Methods for details). (**B** and **C**) Representative images showing the expression of the HA-tagged G12D receptor in the ARC of POMC-G12D mice (mouse age: 20 weeks). Scale bars, 100 μm. (**D**) Acute food intake measurements. POMC-G12D mice and control littermates received a single dose of CNO (5 mg/kg, ip), followed by the monitoring of food intake at the beginning of the dark cycle for 4 hours (hrs). (**E**) Food intake measurements over several days. Mice were injected with a single dose of CNO (5 mg/kg, ip) at the beginning of the dark cycle, followed by the measurement of food intake over a 3-day period. (**F** and **G**) TEE (F) and RER (G) measurements carried out with POMC-G12D mice and control littermates after acute CNO treatment (5 mg/kg, ip). (**H** and **I**) Blood glucose (H) and intraperitoneal GTT (I) measurements. Mice were injected with CNO alone (5 mg/kg, ip) (H) or with CNO and glucose (I), followed by the monitoring of blood glucose levels. Data shown in [(D) to (I)] were obtained with 8- to 14-week-old male mice (*n* = 6 to 8 per group). Data are given as the means ± SEM. *P < 0.05, as compared with the corresponding control group (two-way repeated measures ANOVA, followed by Šídák’s multiple comparisons test). ns, no statistically significant difference; 3V, third ventricle.

Specifically, we crossed POMC-Cre mice (*29*) with *Rosa26-LSL-G12D-IRES-GFP* mice (abbreviated name: LSL-G12D mice) (*24*). This mating scheme yielded POMC-Cre LSL-G12D mice selectively expressing G12D in POMC neurons (abbreviated name: POMC-G12D mice) and control LSL-G12D littermates lacking the Cre transgene (Fig. 1A). Most POMC-expressing cell bodies are located in the ARC (*30*–*32*). We confirmed the selective expression of G12D in the ARC of POMC-G12D mice by detecting the hemagglutinin (HA) epitope tag that had been fused to the N terminus of G12D (Fig. 1, A to C). Colocalization studies showed that 50.5 ± 5.4% of ARC POMC neurons expressed the G12D receptor (Fig. 1C) (see Materials and Methods for details).

POMC neurons are direct targets of anorectic and glucoregulatory hormones and circulating factors including leptin (*31*) and insulin (*33*). Chemogenetic (*34*) and optogenetic (*35*) approaches have demonstrated that stimulation of POMC neurons results in reduced food intake mediated by the melanocortin system. Prompted by these studies, we first examined whether G12D activation in POMC neurons affected feeding behavior. Acute treatment of POMC-G12D mice (males with free access to regular chow) with a single dose of CNO [5 mg/kg, intraperitoneally (ip)] did not affect food intake during the first 24 hours following CNO injection (Fig. 1, D and E). However, at 48 hours after CNO administration, POMC-G12D mice showed a significant reduction in food intake, as compared with their control littermates (Fig. 1E), indicative of a delayed anorectic effect induced by G12D signaling. Indirect calorimetry studies did not reveal any significant differences in the total energy expenditure (TEE) and respiratory exchange ratio (RER) between the two groups of mice (Fig. 1, F and G) under these experimental conditions. Moreover, body weight was essentially unchanged in both mouse cohorts at the end of the 72-hour food intake experiment (% change in body weight: control mice, −1.7 ± 0.5%; POMC-G12D mice: −1.7 ± 0.7%).

POMC neurons are also known to play a key role in regulating glucose homeostasis (*36*–*38*). We found that acute CNO treatment (5 mg/kg, ip) of POMC-G12D mice had no significant effect on blood glucose levels or glucose tolerance [intraperitoneal glucose tolerance test (GTT)] (Fig. 1, H and I). Together, these data suggest that acute activation of G_12/13_ signaling in POMC neurons results in a delayed reduction in food intake but has no significant effect on blood glucose levels, glucose tolerance, and energy expenditure.

### Chronic G12D activation in POMC neurons does not affect energy homeostasis

We next investigated whether chronic activation of G12D in POMC neurons led to changes in energy and glucose homeostasis. For these studies, we used DCZ, a recently developed CNO derivative, as a DREADD agonist (*27*). DCZ is nearly 100 times more potent than CNO and easily penetrates the blood-brain barrier (*27*, *39*). To chronically treat POMC-G12D mice and their control littermates with DCZ, we added DCZ to the drinking water at a concentration of 10 μg/ml (*40*).

**Fig. 2.**
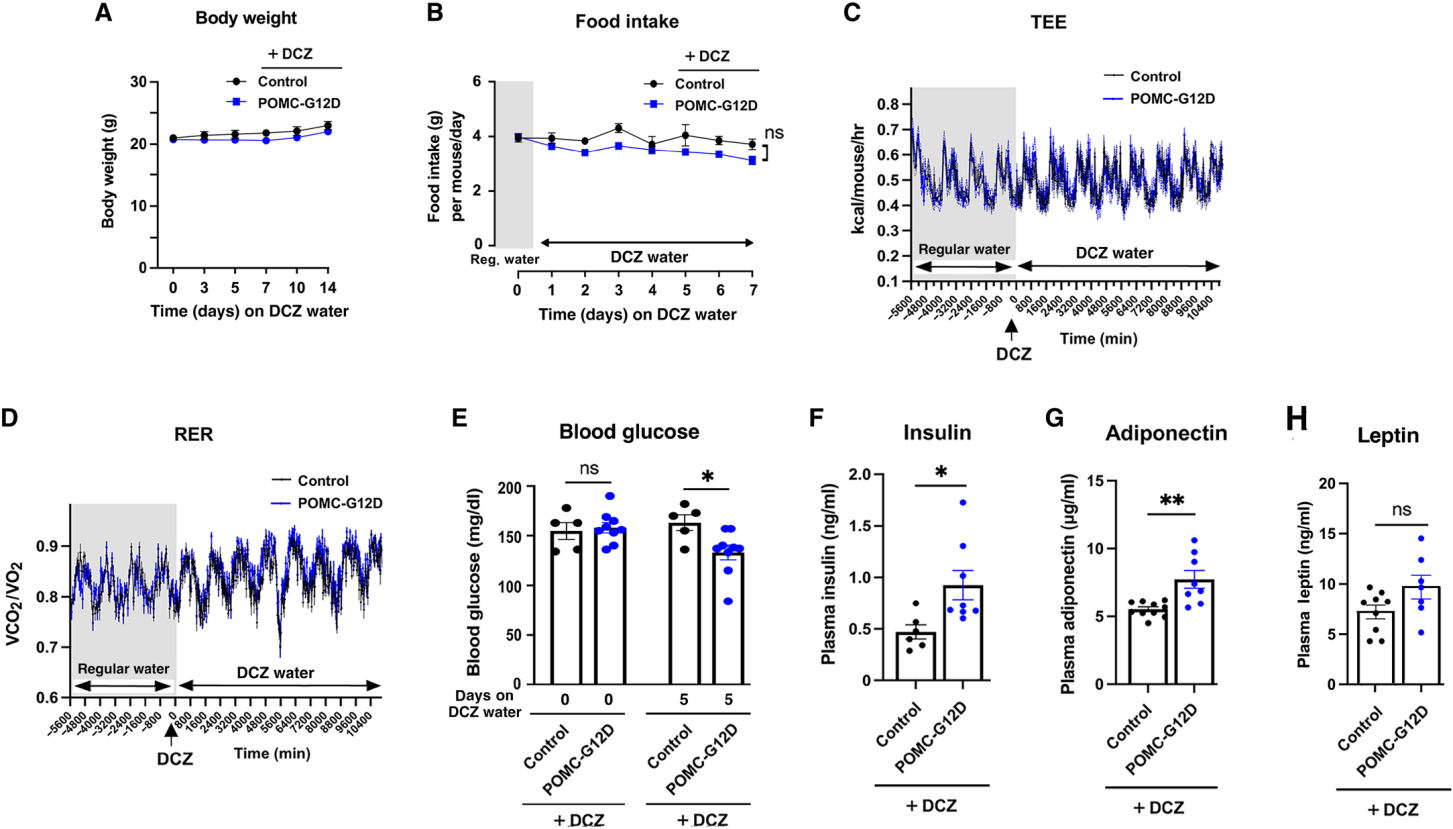
Chronic activation of G12D expressed by POMC neurons leads to various metabolic changes in vivo. All studies were carried out with male mice maintained on regular chow. (**A** and **B**) Body weight (A) and food intake (B) measurements during the chronic treatment of POMC-G12D mice and control littermates with DCZ. Mice consumed drinking water containing DCZ (10 μg/ml) (DCZ water) for 2 weeks (A) or 1 week (B). (**C** and **D**) TEE (C) and RER (D) measurements carried out with POMC-G12D mice and control littermates maintained on regular drinking water for 3 to 4 days, followed by treatment with DCZ water for up to 7 days. (**E**) Blood glucose levels are reduced in POMC-G12D mice consuming DCZ water. Blood was collected between 4 and 5 p.m. Mice had free access to food. (**F** to **H**) Plasma insulin (F), adiponectin (G), and leptin (H) levels of POMC-G12D mice maintained on DCZ water for 2 weeks. The data in [(E) to (H)] were obtained with mice maintained on DCZ water for 2 weeks. Data are given as the means ± SEM (*n* = 6 to 10 per group; mouse age: 8 to 9 weeks). *P < 0.05 and **P < 0.01, as compared with the corresponding control group [two-way repeated measures ANOVA, followed by Šídák’s multiple comparisons test (A) to (E); unpaired *t* test (F) to (H)].

Because long-term activation of ARC POMC neurons causes changes in feeding behavior (*34*), we maintained POMC-G12D mice and their control littermates (males) on DCZ drinking water for up to 2 weeks. The two groups of mice showed similar body weights during the DCZ treatment period (Fig. 2A). Similarly, food intake, TEE, and RER did not differ significantly between POMC-G12D mice and their control littermates maintained on DCZ water (Fig. 2, B to D). These data indicate that chronic activation of G_12/13_ signaling in POMC neurons has no significant effect on body weight and energy homeostasis.

### Chronic G12D activation in POMC neurons causes improved glucose homeostasis

**Fig. 3.**
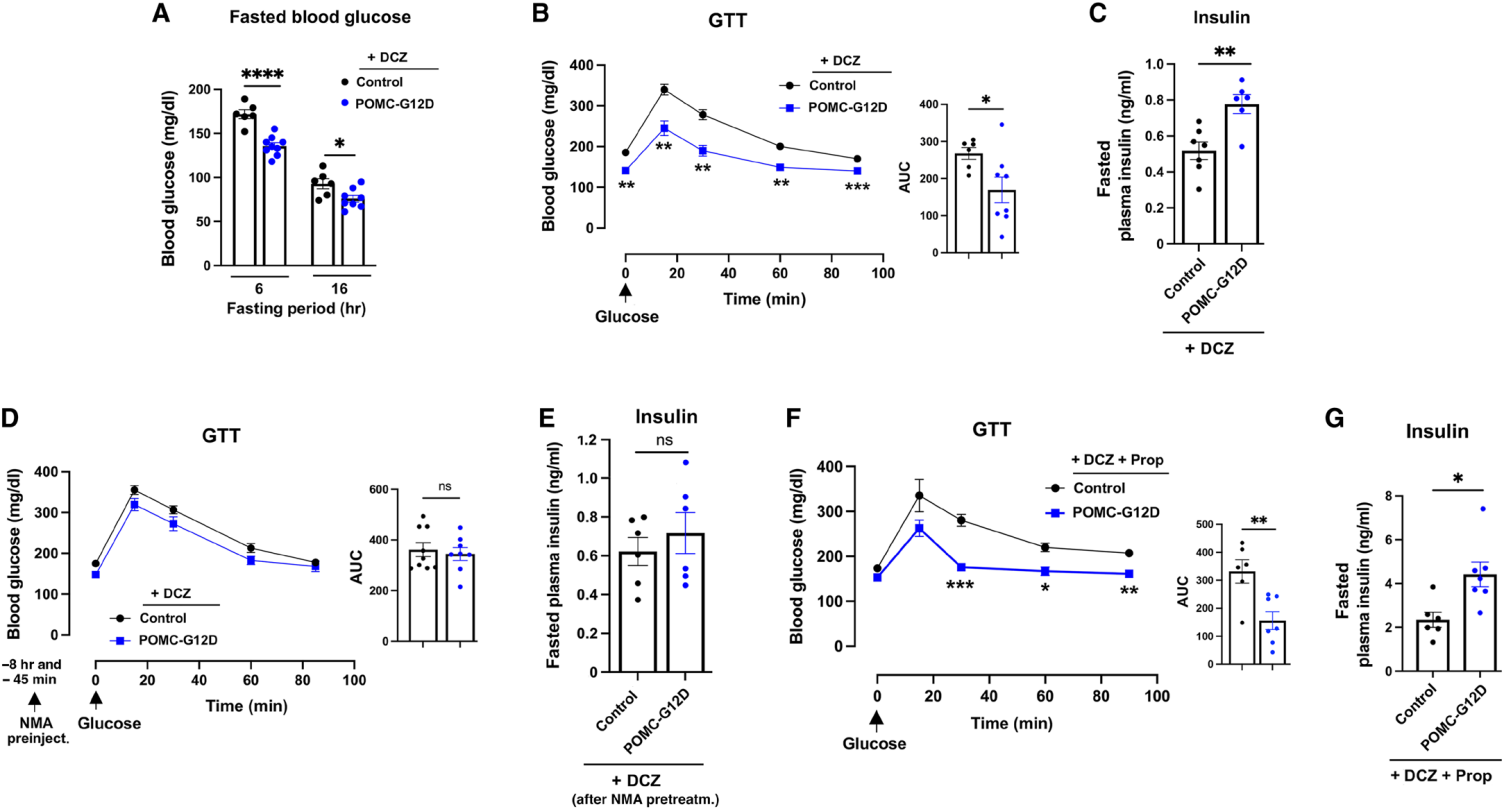
Activation of G12D signaling in POMC neurons improves glucose homeostasis and insulin secretion by enhancing vagal outflow. All studies were carried out with POMC-G12D mice and control littermates (males) consuming regular chow. All mice were maintained on DCZ drinking water (10 μg/ml) for 2 weeks before metabolic measurements. (**A**) Fasting blood glucose levels. (**B**) GTT (1.5 g glucose/kg, ip). (**C**) Fasting plasma insulin levels (6-hour fast). AUC, area under the curve. (**D**) GTT after pretreatment with NMA. POMC-G12D and control mice consuming DCZ water received two doses of NMA (5 mg/kg, ip), as indicated, followed by a GTT (1.5 g glucose/kg, ip). (**E**) Fasting plasma insulin levels (6-hour fast) of POMC-G12D and control mice maintained on DCZ water after NMA treatment (5 mg/kg, ip) [see (D) for details]. (**F**) GTT carried out with POMC-G12D and control mice after consumption of DCZ water containing propranolol (0.5 mg/ml). (**G**) Fasting plasma insulin levels (6-hour fast) of POMC-G12D and control mice maintained on DCZ water containing propranolol (0.5 mg/ml). The data shown in [(A) to (E)] were generated using 8- to 9-week-old mice. Results presented in [(F) and (G)] were obtained with 18-week-old mice. Data are represented as the means ± SEM (*n* = 6 to 9 per group). *P < 0.05, **P < 0.01, ***P < 0.01, and ****P < 0.0001, as compared with the corresponding control group [two-way ANOVA, followed by Šídák’s multiple comparisons test (A); two-way repeated measures ANOVA, followed by Šídák’s multiple comparisons test (B), (D), and (F); unpaired *t* test (C), (E), and (G)]. Prop, propranolol.

POMC-G12D mice (males) consuming DCZ drinking water showed a significant decrease in fed and fasting blood glucose levels, as compared to their control littermates (Figs. 2E and 3A). Although the two cohorts of mice did not differ in body weight when maintained on DCZ water (Fig. 2A), chronic DCZ treatment of POMC-G12D mice resulted in a marked improvement in glucose tolerance (Fig. 3B). At the same time, plasma insulin and adiponectin levels were elevated in DCZ-treated POMC-G12D mice (Figs. 2, F and G, and 3C). Plasma leptin levels did not differ significantly between the two groups of mice (Fig. 2H).

### DCZ is stable in aqueous solution and causes long-lasting effects

To determine the stability of DCZ in aqueous solution at room temperature (23°C) for up to 4 weeks, we used liquid chromatography–mass spectrometry. Liquid chromatography–mass spectra clearly showed that DCZ dissolved in water (10 μg/ml) was stable at room temperature at all time points examined (1, 2, and 4 weeks, respectively) (fig. S1).

We also wanted to confirm that water consumption was similar between control and POMC-G12D mice. To address this issue, we maintained POMC-G12D mice and control littermates on regular drinking water for 4 days. Regular drinking water was then replaced with DCZ water for the following 4 days. We found that the daily consumption of DCZ water or regular water did not differ significantly between POMC-G12D and control mice (fig. S2).

To confirm that DCZ retained biological activity after a prolonged period of DCZ water consumption, we maintained POMC-G12D mice and control littermates on DCZ drinking water for 1 month. We then subjected the two groups of mice to an intraperitoneal GTT. We found that DCZ-treated POMC-G12D mice showed significantly improved glucose tolerance, as compared to DCZ-treated control littermates (fig. S3). This observation indicates that DCZ retains biological activity in the drinking water after an extended period of time (1 month).

### Female POMC-G12D mice show similar metabolic phenotypes to their male counterparts

We also carried out metabolic studies with female POMC-G12D consuming DCZ drinking water. In general, chronic DCZ treatment of female POMC-G12D mice led to qualitatively similar metabolic changes as observed with their male counterparts (fig. S4). Like male POMC-G12D mice, female POMC-G12D mice consuming DCZ drinking water showed no significant differences in body weight and food intake (fig. S4, A and B) but greatly improved glucose tolerance, as compared to their control littermates (fig. S4C). Moreover, as observed with their male counterparts, chronic DCZ treatment of female POMC-G12D mice led to significant increases in plasma insulin and adiponectin levels (fig. S4, D and F) but did not cause a significant change in plasma leptin levels (fig. S4E).

### G12D-mediated beneficial metabolic effects are abolished by *N*-methylatropine

As outlined in the previous paragraphs, POMC-G12D mice consuming DCZ drinking water showed marked improvements in glucose tolerance and elevated plasma insulin levels, as compared to their control littermates. Because parasympathetic outflow to the endocrine pancreas strongly promotes insulin secretion when blood glucose levels are high (*41*–*43*), we speculated that the improvements in glucose homeostasis displayed by DCZ-treated POMC-G12D mice might involve enhanced efferent vagal activity. To test this hypothesis, we treated POMC-G12D mice consuming DCZ drinking water with *N*-methylatropine (NMA), followed by an intraperitoneal GTT. NMA inhibits vagal efferent activity by blocking muscarinic acetylcholine receptors expressed by peripheral organs or tissues innervated by parasympathetic nerves (*44*, *45*). Specifically, mice received two intraperitoneal injections of NMA (5 mg/kg per injection). Dose 1 was given at 10 a.m., and dose 2 was administered at 5 p.m. (*44*, *45*). Forty-five minutes after the second NMA injection, mice received an intraperitoneal glucose bolus (1.5 g/kg). In agreement with our hypothesis, NMA pretreatment of POMC-G12D mice maintained on DCZ water abolished the improvement in glucose tolerance observed with the DCZ-treated POMC-G12D mice in the absence of NMA (Fig. 3, B and D). Moreover, following NMA administration, fasting plasma insulin levels were no longer elevated in POMC-G12D mice consuming DCZ water, as compared with DCZ-treated control littermates (Fig. 3, C and E).

It is well known that changes in sympathetic outflow can also modulate peripheral functions involved in the regulation of glucose homeostasis (*46*). To explore a potential role of altered sympathetic outflow in the metabolic improvements observed with DCZ-treated POMC-G12D mice, we maintained POMC-G12D mice and control littermates on DCZ drinking water containing propranolol (0.5 mg/ml) (*47*) for 2 weeks. Propranolol blocks β-adrenergic receptors that mediate many of the important metabolic actions triggered by stimulation of the peripheral sympathetic nervous system (*48*). We found that propranolol treatment had no significant effect on the improved glucose tolerance displayed by DCZ-treated POMC-G12D mice (Fig. 3F), suggesting that enhanced sympathetic outflow does not contribute to this effect. Similarly, propranolol administration had no significant effect on the elevated plasma insulin observed with POMC-G12D mice consuming DCZ water (Fig. 3G).

### Chronic G12D activation in POMC neurons ameliorates obesity-induced metabolic deficits

**Fig. 4.**
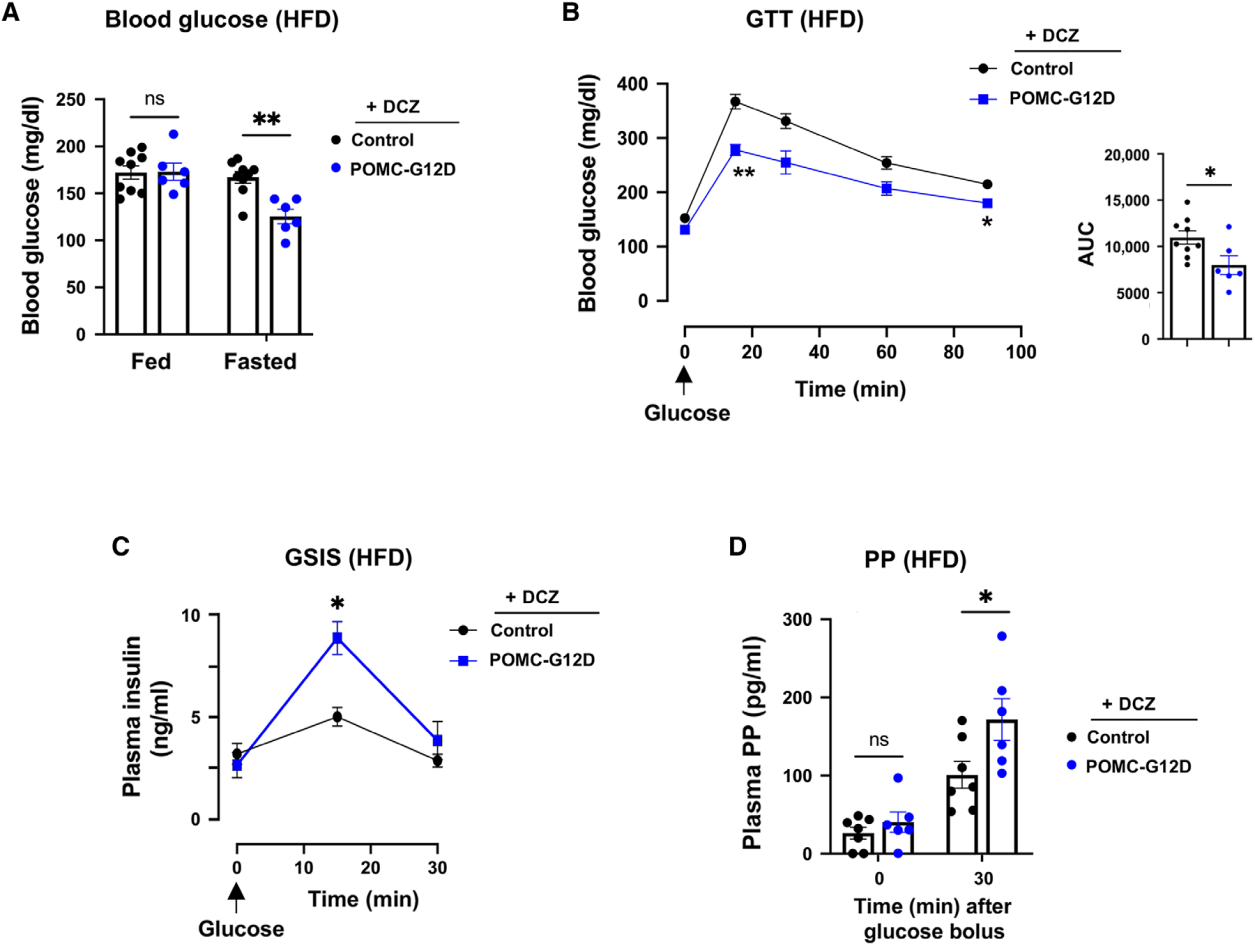
Chronic G12D activation in POMC neurons ameliorates deficits in glucose homeostasis caused by a calorie-rich diet. All studies were carried out with POMC-G12D mice and control littermates (males) consuming an HFD for 12 to 13 weeks. During the HFD feeding period, all mice were maintained on DCZ drinking water (10 μg/ml). (**A**) Fed and fasting blood glucose levels of POMC-G12D and control mice maintained on an HFD for 12 weeks. (**B**) GTT (1 g glucose/kg, ip) performed with POMC-G12D and control mice maintained on an HFD for 13 weeks. (**C** and **D**) Glucose (1 g/kg, ip)–induced increases in plasma insulin (C) and plasma PP (D) levels of POMC-G12D and control mice maintained on an HFD for 13 weeks. HFD feeding was initiated when mice were 20 weeks old. Data are represented as the means ± SEM (*n* = 6 to 9 per group). *P < 0.05 and **P < 0.01, as compared with the corresponding control group [two-way ANOVA, followed by Šídák’s multiple comparisons test for (A) to (D); unpaired *t* test (B); AUC data)].

We next examined whether chronic activation of G12D signaling in POMC neurons could counteract the deficits in glucose homeostasis caused by the prolonged consumption of a high-fat diet (HFD). Specifically, we maintained POMC-G12D mice and control littermates (20-week-old males) on an HFD for 8 to 12 weeks. During HFD feeding, both mouse cohorts consumed DCZ drinking water. Under these experimental conditions, the two groups of mice displayed similar increases in body weight gain and adiposity (fig. S5, A and B). However, POMC-G12D mice showed significantly reduced fasting blood glucose levels (Fig. 4A; 16-hour overnight fast) and improved glucose tolerance (Fig. 4B). When challenged with a glucose bolus (1 g/kg, ip), obese POMC-G12D mice consuming DCZ water released significantly more insulin than their obese control littermates [GSIS (glucose-stimulated insulin secretion); Fig. 4C], explaining why the DCZ-consuming obese POMC-G12D mice showed improved glucose tolerance.

The data shown in Fig. 3 (B to E) suggested that chronic activation of G12D signaling in POMC neurons stimulates parasympathetic outflow in lean mice, leading to enhanced insulin secretion and improved glucose tolerance. Because plasma pancreatic polypeptide (PP) levels are considered a useful surrogate marker for pancreatic vagal tone (*49*–*51*), we determined plasma PP levels before glucose administration (baseline levels) and after injection of a glucose bolus into obese POMC-G12D mice and control littermates consuming DCZ drinking water. While baseline plasma PP levels were not significantly different between the two groups of mice, injection of an intraperitoneal glucose bolus (1 g/kg) into obese POMC-G12D mice caused a significant increase in plasma PP levels, as compared with control littermates treated in the same fashion (Fig. 4D). These results indicate that chronic activation of G_12/13_ signaling in POMC neurons greatly improves glucose tolerance in obese mice, most likely by enhancing parasympathetic flow to the endocrine pancreas.

### Elevated plasma adiponectin results from reduced sympathetic flow to adipose tissues

Another notable feature displayed by both male and female POMC-G12D mice consuming DCZ water was a significant increase in plasma adiponectin levels (Fig. 2G and fig. S4F). To further explore the cellular mechanisms underlying this phenotype, we carried out additional in vitro and in vivo studies.

**Fig. 5.**
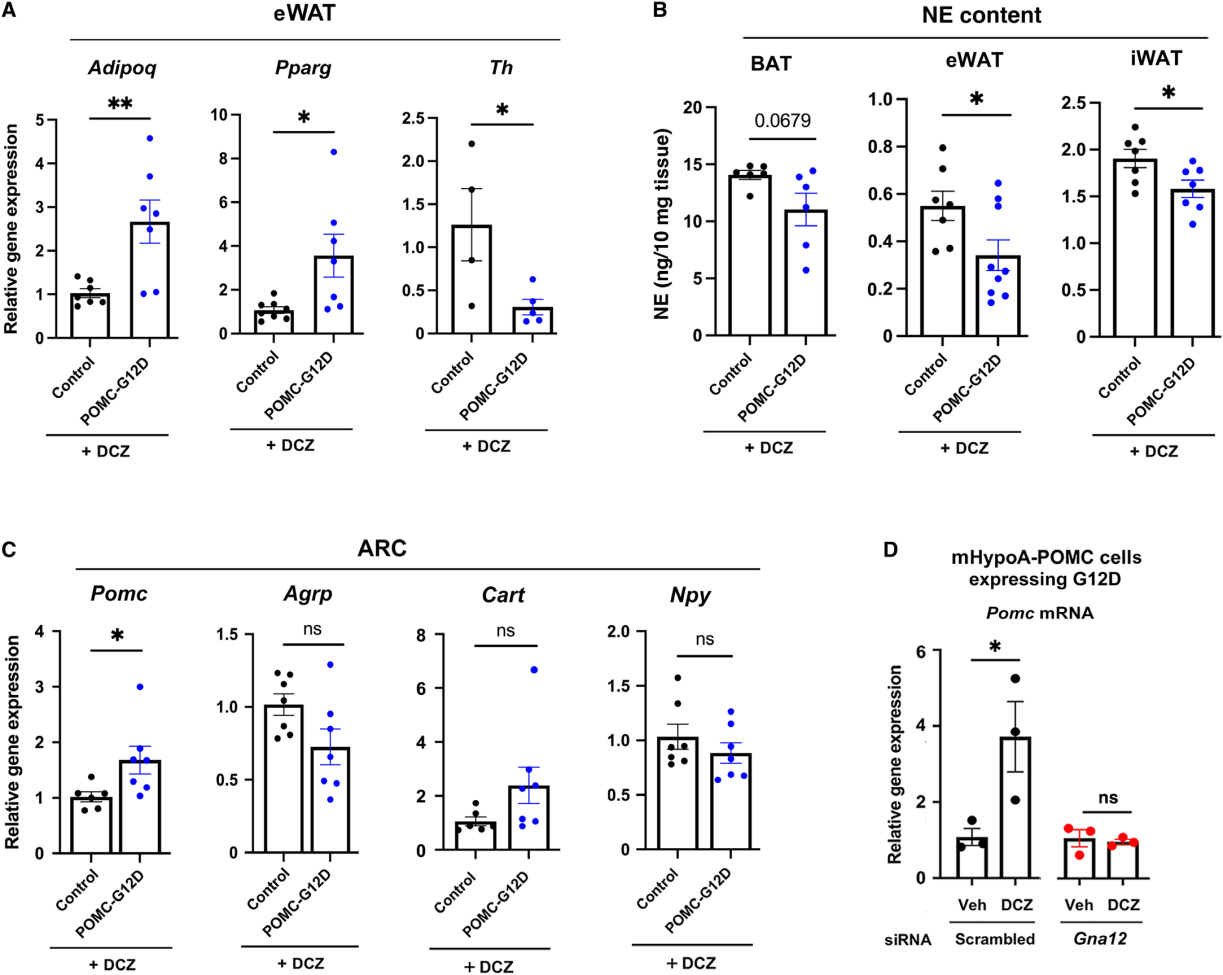
G12D-mediated signaling decreases markers of sympathetic tone in fat tissues and enhances *Pomc* expression. (**A**) qRT-PCR analysis of *Adipoq*, *Pparg*, and *Th* mRNA levels in eWAT obtained from POMC-G12D mice and control littermates maintained on DCZ drinking water. (**B**) NE content of BAT, eWAT, and iWAT of POMC-G12D and control mice chronically treated with DCZ water. (**C**) qRT-PCR analysis of *Pomc*, *Agrp*, *Cart*, and *Npy* transcript levels in ARC mRNA prepared from POMC-G12D mice and control littermates maintained on DCZ water. At the time of tissue collection, mice had been drinking DCZ water for 4 weeks. (**D**) G12D activation promotes *Pomc* gene expression in mHypoA-POMC cells expressing the G12D designer receptor. The treatment of these cells with DCZ (50 nM) for 3 hours caused a robust increase in *Pomc* gene expression, as demonstrated via qRT-PCR. This effect was abolished after treatment of cells with siRNA for *Gna12* (encoded protein: Gα_12_). Data from three independent experiments are shown. Data shown in [(A) to (C)] were generated using tissues/organs from female mice (age: ~20 weeks). Data are given as the means ± SEM (*n* = 4 to 7 per group). *P < 0.05 and **P < 0.01, as compared with the corresponding control group (unpaired *t* test). Numbers above horizontal bars represent *P* values. Veh, vehicle.

Because white adipose tissue (WAT) is the major source of circulating adiponectin (*52*), we prepared RNA from different WAT depots and studied the expression levels of key genes involved in WAT function (fig. S6). Quantitative reverse transcription polymerase chain reaction (qRT-PCR) studies revealed a significant increase in *Adipoq* (encoded protein: adiponectin) and *Pparg* mRNA expression levels (fig. S6 and Fig. 5A) in epidydimal WAT (eWAT) from POMC-G12D mice (males and females) maintained on DCZ drinking water, as compared to control littermates treated in an identical fashion. Because enhanced PPARγ (peroxisome proliferator–activated receptor γ) signaling promotes the production of adiponectin (*53*, *54*), the observed increase in *Adipoq* expression is most likely secondary to enhanced Pparγ signaling.

WAT is abundantly innervated by sympathetic nerves but receives little or no input from the parasympathetic nervous system [reviewed in (*55*)]. Published work (*56*, *57*) has demonstrated that decreased sympathetic flow to mouse adipose tissue depots results in elevated circulating adiponectin levels. Prompted by this observation, we investigated whether POMC-G12D mice maintained on DCZ water showed altered sympathetic activity in WAT. Specifically, we measured WAT transcript levels of the *Th* gene (encoded protein: tyrosine hydroxylase) and WAT norepinephrine (NE) content, two commonly used surrogate markers of sympathetic tone. Adipose tissue weights did not differ significantly between the two groups of mice (DCZ-treated POMC-G12D mice versus DCZ-treated control littermates) (fig. S7.). We found that *Th* mRNA levels were markedly reduced in WAT (eWAT) of POMC-G12D mice consuming DCZ water (Fig. 5A). Moreover, NE levels were significantly decreased in both eWAT and inguinal WAT [iWAT; but not in brown adipose tissue (BAT)] of DCZ-treated POMC-G12D mice (Fig. 5B). These data strongly suggest that chronic activation of G_12/13_ signaling in POMC neurons reduces sympathetic flow to adipose tissue depots. It is likely that this effect contributes to the marked increase in circulating adiponectin levels displayed by DCZ-treated POMC-G12D mice (*56*, *57*).

### Increased plasma adiponectin levels contribute to improved glucose tolerance

It is well known that adiponectin improves glucose tolerance and insulin sensitivity by acting on AdipoR1 and AdipoR2 receptors expressed by insulin-sensitive tissues (*58*). To explore the physiological relevance of the increase in plasma adiponectin levels displayed by DCZ-treated POMC-G12D mice, we took advantage of the availability of an adiponectin receptor antagonist (Chex-DSer-8 antagonist/ADP 400), which blocks both AdipoR1 and AdipoR2 receptors (*59*–*62*). Specifically, we performed an intraperitoneal GTT with ADP 400–preinjected POMC-G12D and control mice that had been maintained on DCZ water for 2 weeks. Before glucose treatment, mice received two injections of ADP 400 [1 mg/kg, subcutaneously (sc); 1 and 7 hours before glucose administration]. Notably, under these experimental conditions, ADP 400–treated POMC-G12D and control mice did no longer show a significant difference in glucose tolerance (fig. S8). This observation supports the concept that the G12D-mediated increase in plasma adiponectin levels contributes to the improved glucose tolerance displayed by DCZ-treated POMC-G12D mice.

### G12D activation increases *Pomc* mRNA levels in the ARC and mHypoA-POMC cells

We next investigated whether chronic activation of G_12/13_ signaling in POMC neurons affected *Pomc* gene expression in the ARC. qRT-PCR studies showed that *Pomc* transcript levels were significantly increased in the ARC of POMC-G12D mice maintained on DCZ drinking water, as compared to control littermates not expressing the G12D designer receptor (Fig. 5C). Chronic DCZ treatment of POMC-G12D mice had no significant effect on the mRNA levels of several other metabolically important neuropeptides expressed by distinct sets of ARC neurons (*Agrp*, *Cart*, and *Npy*) (Fig. 5C).

In addition, we used a G12D-encoding adenovirus to express the G12D designer receptor in a POMC neuron–derived cell line (mHypoA-POMC/GFP-2 cells; short name: G12D-mHypoA-POMC cells) (fig. S9A). This cell line has been successfully used in the past as an in vitro model system that mimics many of the key features of native POMC neurons including the regulation of *Pomc* expression levels and α-MSH secretion in response to anorectic hormones (*63*). We found that DCZ treatment (50 nM) of G12D-mHypoA-POMC cells caused a significant increase in *Pomc* gene expression (Fig. 5D), in agreement with the expression data shown in Fig. 5C. This effect was abolished after small interfering RNA (siRNA)–mediated knockdown of *Gna12* expression (Fig. 5D and fig. S9, B to D), indicating that this G12D response was mediated by Gα_12_ signaling.

### Chronic G12D activation in POMC neurons enhances leptin effects

**Fig. 6.**
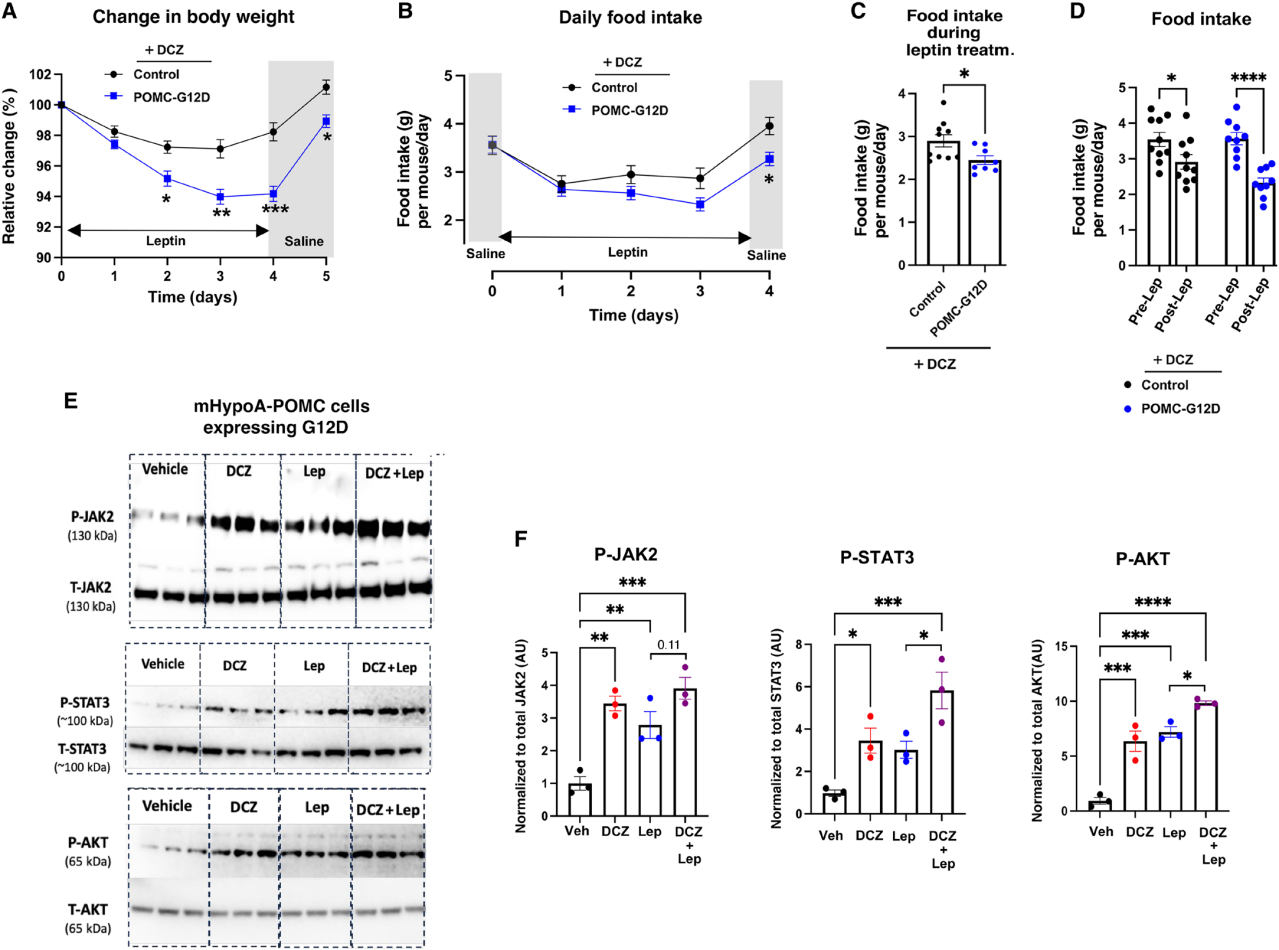
Activation of G12D signaling in POMC neurons strongly enhances leptin effects in vivo and in vitro. All in vivo studies were carried out with male mice consuming regular chow. (**A** and **B**) Leptin-induced decreases in body weight (A) and food intake (B) are more pronounced in POMC-G12D mice maintained on DCZ water. POMC-G12D mice and control littermates were initially subjected to saline injections for three consecutive days (twice daily), followed by treatment with leptin for three consecutive days (2.5 mg/kg, ip, twice daily). All mice were maintained on DCZ water (10 μg/ml) during the course of this study. (**C**) Average daily food intake per mouse during the 3-day leptin treatment period. (**D**) Daily food intake before leptin administration [day 0 in (B)] and after 3 days of leptin treatment. Data shown in (A) to (D) were obtained with 11-week-old mice (*n* = 8 to 10 per group). (**E**) Immunoblotting experiments. G12D-expressing mHypoA-POMC cells were incubated with either vehicle, DCZ (50 nM), leptin (100 nM), or both DCZ and leptin for 15 min. Subsequently, cell lysates were subjected to Western blotting experiments using antibodies against the indicated proteins. Three independent experiments were performed. (**F**) Quantification of the Western blotting data shown in (E). Data are given as the means ± SEM. *P < 0.05, **P < 0.01, ***P < 0.001, and ****P < 0.0001, as compared with the corresponding control group [two-way repeated measures ANOVA, followed by Šídák’s multiple comparisons test (A) and (B); unpaired *t* test (C); two-way ANOVA, followed by Šídák’s multiple comparisons test (D); one-way ANOVA, followed by Tukey’s multiple comparison test (F)]. Numbers above horizontal bars represent *P* values. AU, arbitrary units; Lep, leptin.

Leptin, a key regulator of the function of POMC neurons, binds to and activates leptin receptors expressed by POMC neurons, leading to reduced food intake and various other beneficial metabolic changes (*29*, *64*, *65*). For example, Berglund *et al.* (*38*) previously demonstrated that reexpression of the leptin receptor selectively in POMC neurons of mice unable to express the leptin receptor resulted in improved blood glucose homeostasis independent of changes in body weight. Thus, we next investigated whether activation of G_12/13_ signaling in POMC neurons affected the metabolic effects of leptin. Initially, we injected POMC-G12D mice and control littermates consuming DCZ drinking water with leptin for three consecutive days (two intraperitoneal injections per day; 2.5 mg/kg per injection). As expected, leptin treatment suppressed food intake in control mice lacking the G12D receptor, resulting in a reduction in body weight and food intake (Fig. 6, A and B). These leptin effects were significantly enhanced in POMC-G12D mice consuming DCZ drinking water (Fig. 6, A to D).

### G_12/13_ signaling in mHypoA-POMC cells activates similar signaling pathways to leptin

At the cellular level, leptin stimulates the Janus kinase 2 (JAK2)-signal transducers and activators of transcription 3 (STAT3) signaling pathway, leading to the activation of the *Pomc* promoter (*66*). Many studies have shown that stimulation of the JAK2-STAT3 signaling cascade plays a key role in mediating the beneficial metabolic effects of leptin (*66*–*68*). Previous work has demonstrated that activation of G_12/13_ signaling can also stimulate JAK2-STAT3 signaling in various cell types via activation of RhoA or other G proteins of the Rho family (*69*, *70*).

To further explore leptin/G12D–mediated signaling, we investigated leptin/G12D–induced signaling pathways in G12D-mHypoA-POMC cells. We initially demonstrated that leptin treatment (100 nM) of G12D-mHypoA-POMC cells stimulated the phosphorylation of JAK2 and STAT3 (Fig. 6, E and F). In agreement with findings obtained with other cell types (*69*, *70*), incubation of G12D-mHypoA-POMC cells with DCZ (50 nM) yielded similar results (Fig. 6, E and F). Cotreatment with leptin and DCZ stimulated STAT3 phosphorylation to a significantly greater extent than treatment with leptin alone (Fig. 6, E and F).

Besides promoting JAK2-STAT3 signaling, leptin also activates the phosphatidylinositol 3-kinase/Akt signaling pathway in POMC neurons, an effect that contributes to the beneficial metabolic actions of leptin (*65*, *71*, *72*). As expected, leptin treatment (100 nM) of G12D-mHypoA-POMC cells strongly stimulated the formation of p-Akt (Thr^308^) (Fig. 6, E and F). DCZ (50 nM) treatment of these cells resulted in a very similar response (Fig. 6, E and F). Co-incubation of G12D-expressing cells with leptin and DCZ stimulated the formation of p-Akt to a significantly greater extent than treatment with leptin alone (Fig. 6, E and F).

Treatment of G12D-mHypoA-POMC cells with leptin (100 nM) also caused a significant increase in α-MSH secretion (fig. S9E). In contrast, treatment with DCZ (50 nM) had no significant effect on α-MSH release (fig. S9E), suggesting that the stimulatory effect of G12D on *Pomc* expression (Fig. 5D) does not lead to an acute stimulation of α-MSH secretion. This finding offers a possible explanation for the delayed anorectic effect observed after acute stimulation of G12D in POMC neurons in vivo (Fig. 1E).

Together, these data indicate that stimulation of G_12/13_ signaling in mHypoA-POMC cells activates similar signaling pathways to leptin. This observation can explain why DCZ treatment of POMC-G12D mice enhanced the beneficial metabolic effects of leptin in vivo (Fig. 6, A to D).

### G12D activation increases leptin signaling in ARC POMC neurons

To investigate whether leptin sensitivity is improved in ARC POMC neurons following G12D activation in vivo, we used immunohistochemistry to detect p-STAT3 formation in G12D+ neurons of POMC-G12D mice maintained on DCZ drinking water (fig. S10, B and D). For control purposes, we carried out analogous experiments with DCZ-treated control mice lacking the G12D receptor (fig. S10, A and C). After an overnight fast (16 hours), one group of mice received a single intraperitoneal saline injection, while a second group was treated with a single dose of leptin (5 mg/kg, ip). Brain tissue was collected 1 hour after saline or leptin injections. In saline-injected control mice, neurons displaying p-STAT3 staining were very rare (fig. S10A). In contrast, chronic DCZ treatment of POMC-G12D mice greatly enhanced the number of pSTAT3-positive G12D+ neurons (fig. S10B). As expected, leptin treatment of control mice resulted in a strong p-STAT3 signal in the ARC (fig. S10C). p-STAT3 staining was even more pronounced in ARC POMC neurons of leptin-treated POMC-G12D mice maintained on DCZ water (% G12D+ neurons staining positive for pSTAT3: POMC-G12D mice on DCZ water, 41.9 ± 6.0%; leptin-treated POMC-G12D mice on DCZ water, 57.6 ± 7.1%) (fig. S10D).

### G12D signaling stimulates the Akt signaling cascade in the ARC

**Fig. 7.**
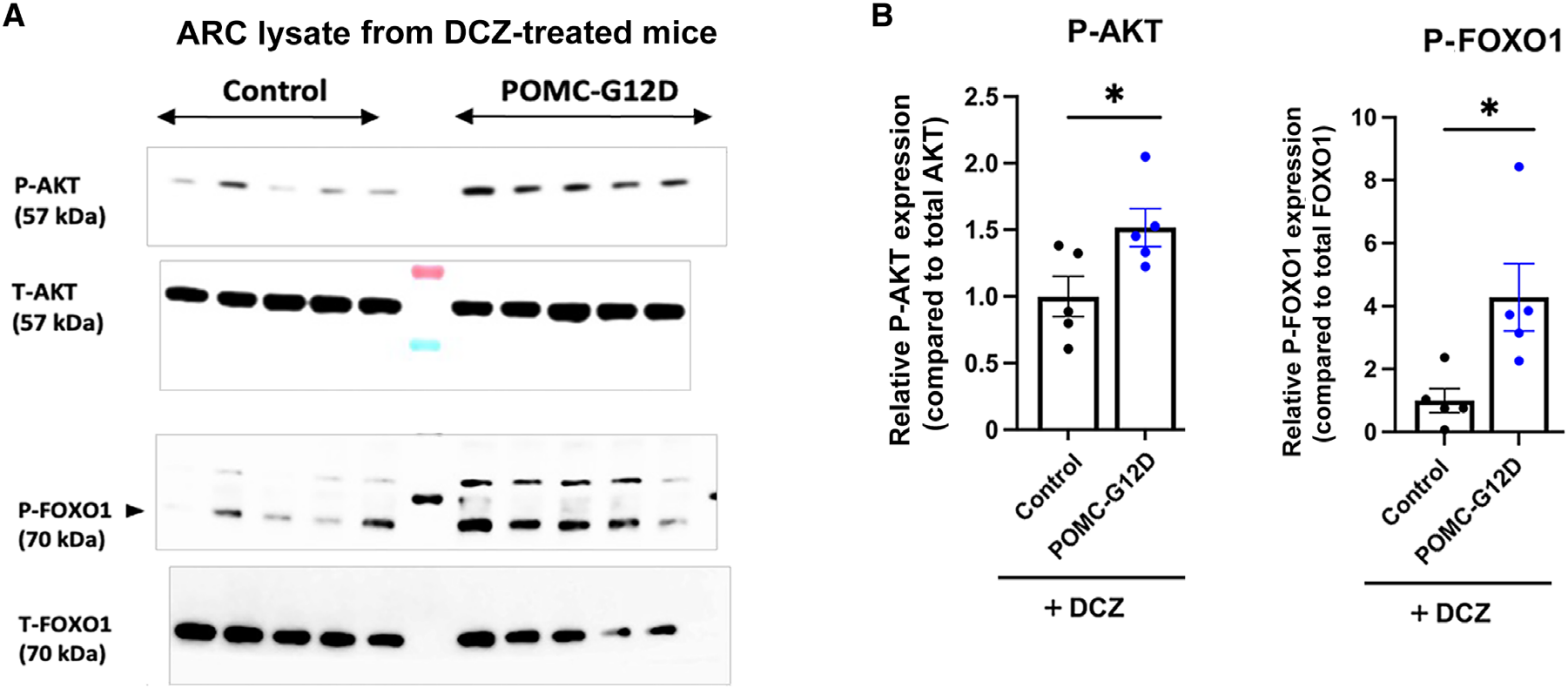
G12D-mediated phosphorylation of Akt and FOXO1 in the ARC of POMC-G12D mice. (**A**) Immunoblotting studies. Lysates were prepared from the mediobasal hypothalamus containing the ARC of POMC-G12D mice and control littermates that had been maintained on DCZ water for 4 weeks (*n* = 5 per group). Protein lysates were subjected to Western blotting studies using the indicated antibodies. (**B**) Quantification of the Western blotting data shown in (A) via densitometry of immunoreactive bands. Experiments were carried out using tissues from 20-week-old mice. Data are given as the means ± SEM. *P < 0.05, as compared with the corresponding control group (unpaired *t* test).

To confirm the physiological relevance of the data obtained with G12D-mHypoA-POMC cells, we carried out immunoblotting studies using tissue lysates prepared from the mediobasal hypothalamus containing the ARC of POMC-G12D mice and control littermates maintained on DCZ drinking water. Consistent with the findings obtained with cultured cells, chronic G12D signaling promoted the formation of p-Akt in the ARC (Fig. 7, A and B). In agreement with this observation, the expression levels of p-FOXO1 and p-GSK3-β, two major cellular targets of activated Akt, were also elevated in the ARC of DCZ-treated POMC-G12D mice (Fig. 7, A and B, and fig. S9, F and G). Because the conversion of FOXO1 to p-FOXO1 stimulates the activity of the *Pomc* promoter (*73*, *74*), these data support the concept that G12D/Akt–mediated phosphorylation of FOXO1 contributes to the increase in *Pomc* expression in the ARC of DCZ-treated POMC-G12D mice (Fig. 5C).

### G_12/13_ signaling does not enhance insulin signaling in mHypoA-POMC cells

To explore the possibility that activation of G_12/13_ signaling enhances insulin signaling in POMC-expressing cells, we treated G12D-expressing mHypoA-POMC cells (G12D-mHypoA-POMC cells) with either DCZ (50 nM) or insulin (100 nM) alone or a mixture of DCZ and insulin (fig. S11). As expected, insulin efficiently stimulated the formation of p-Akt, p-FOXO1, and p-Gsk3β. Like insulin, DCZ alone stimulated the phosphorylation of Akt and FOXO1 (the DCZ-induced increase in p-Gsk3β formation failed to reach statistical significance) (fig. S11). Cotreatment of G12D-mHypoA-POMC cells with insulin and DCZ did not result in a further increase in p-Akt, p-FOXO1, and p-Gsk3β formation, as compared to insulin treatment alone (fig. S11). These data suggest that activation of G_12/13_ signaling does not enhance insulin signaling, at least not in G12D-mHypoA-POMC cells.

### G12D-stimulated ARC Akt signaling does not affect whole-body insulin sensitivity

To determine whether changes in G12D-mediated increases in pAkt activity in the ARC of POMC-G12D mice affected whole insulin sensitivity, we subjected POMC-G12D mice and control littermates maintained on DCZ water for 2 weeks to an insulin tolerance test (fig. S12). We found that insulin sensitivity was not significantly different between the two groups of mice, suggesting that G12D-mediated changes in ARC Akt signaling do not affect whole-body insulin sensitivity under our experimental conditions.

### DCZ, like leptin, leads to the depolarization of G12D-expressing POMC neurons

**Fig. 8.**
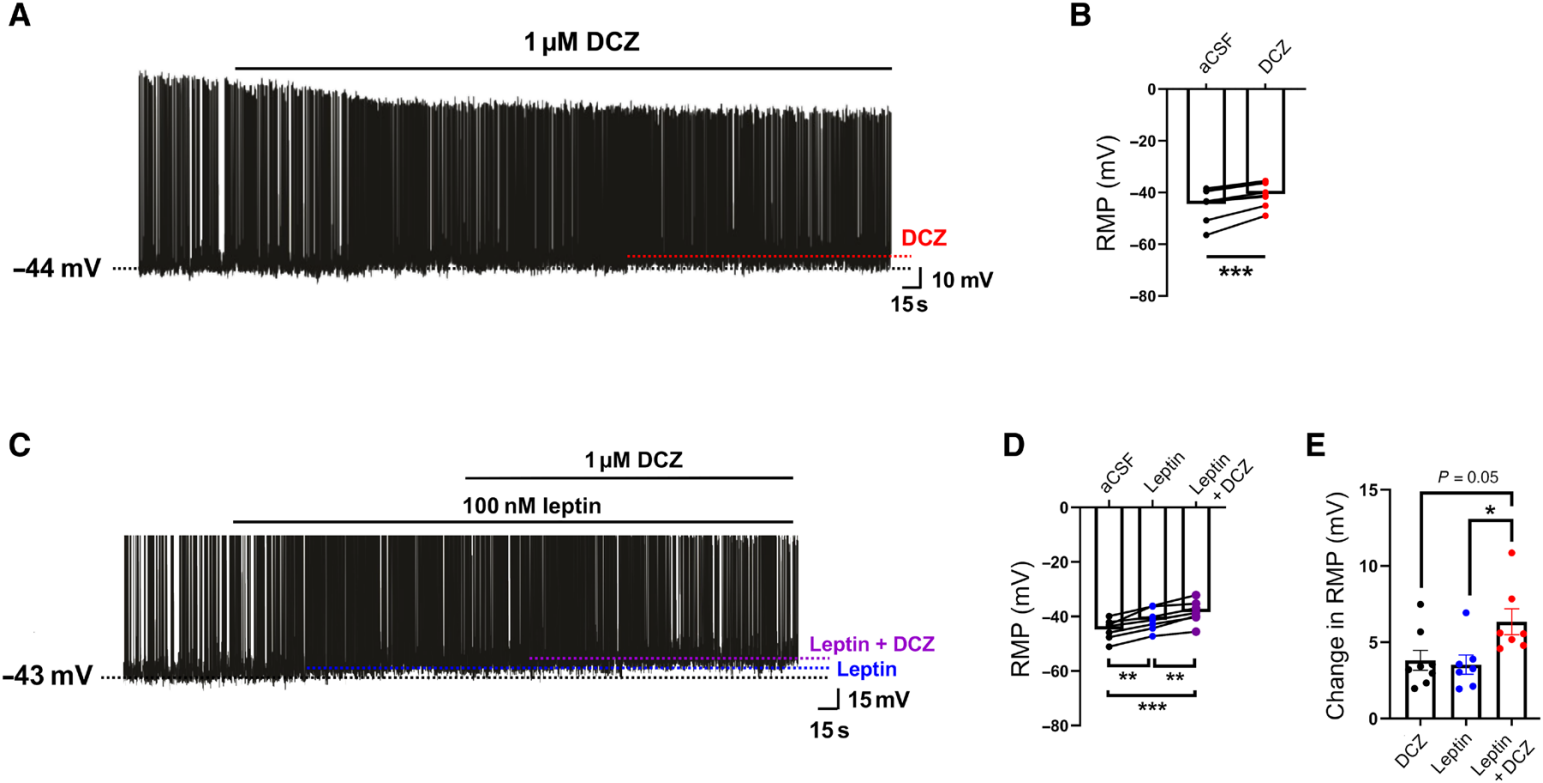
DCZ-induced depolarization of G12D-expressing ARC POMC neurons and synergistic effect of leptin and DCZ. (**A**) Current-clamp recording depicting a characteristic depolarization response of G12D-expressing ARC POMC neurons in the presence of DCZ (1 μM). Black stippled line: RMP in the presence of aCSF (control). Red stippled line: RMP in the presence of DCZ. (**B**) Histogram summarizing the acute effect of DCZ (1 μM) on the membrane potential of POMC neurons (n = 8 neurons). (**C**) Current-clamp record depicting a characteristic depolarization of G12D-expressing ARC POMC neurons by leptin (100 nM), followed by the addition of DCZ (1 μM). Black stippled line: RMP in the presence of aCSF (control). Blue stippled line: RMP in the presence of leptin. Purple stippled line: RMP in the presence of leptin and DCZ. (**D**) Histogram summarizing the effect of leptin (100 nM) and leptin and DCZ (1 μM) on the membrane potential of G12D POMC neurons (*n* = 7). (**E**) Histogram summarizing the change in RMP of G12D POMC neurons caused by bath application of DCZ alone (*n* = 8), leptin alone (*n* = 7), or a mixture of DCZ + leptin (*n* = 7) (*n* refers to the number of neurons examined). Experiments were carried out using tissues from POMC-G12D mice injected with the AAV-DIO-mCherry virus into the ARC (mouse age: 20 weeks). Data are expressed as the means ± SEM. **P < 0.01 and ***P < 0.001 [paired *t* test (B) and one-way ANOVA, followed by Tukey’s multiple comparisons test].

In addition to examining G12D-stimulated signaling pathways, we also carried out a series of electrophysiological studies. Initially, we examined the response of G12D-expressing ARC POMC neurons to bath application of DCZ (1 μM) ex vivo using whole-cell patch-clamp electrophysiology. We found that DCZ treatment depolarized the resting membrane potential (RMP) in these neurons (Fig. 8, A and B).

Prompted by the outcome of the biochemical and physiological studies, we next examined whether G_12/13_ and leptin signaling affects the electrophysiological properties of ARC POMC neurons in a synergistic fashion. Specifically, we performed whole-cell patch-clamp recordings of G12D-expressing ARC POMC neurons treated with leptin (100 nM), followed by bath application of a mixture of leptin (100 nM) and DCZ (1 μM). As reported previously (*71*, *75*), leptin treatment depolarized about one-third of POMC neurons (7 of 20 neurons) (Fig. 8C). Cotreatment of the seven leptin-sensitive POMC neurons expressing G12D with leptin and DCZ further increased the magnitude of the leptin effect (Fig. 8, C and D). Coapplication of DCZ and leptin resulted in an enhanced change in the RMP of POMC neurons, as compared to the corresponding changes obtained with DCZ or leptin alone (Fig. 8, C and E). This observation is in good agreement with the outcome of the biochemical and physiological studies described in the previous paragraphs.

### Chronic DCZ treatment of POMC-G12D mice does not affect corticosterone levels

Adrenocorticotropic hormone is synthesized by and secreted from corticotropic cells of the anterior pituitary gland (*76*). Adrenocorticotropic hormone, which is derived from POMC by enzymatic cleavage, stimulates the production and release of corticosterone from the adrenal cortex (*76*). Besides other actions, corticosterone plays an important role in the regulation of glucose homeostasis (*77*). To rule out the possibility that DCZ treatment of POMC-G12D mice caused changes in plasma corticosterone levels, we measured plasma corticosterone in POMC-G12D mice and control littermates consuming DCZ drinking water. We found that plasma corticosterone levels were similar in both groups of mice, either before DCZ treatment or 2 weeks after DCZ water exposure (fig. S13). These data indicated that changes in the activity of the pituitary-adrenal axis do not contribute to the metabolic phenotypes displayed by the DCZ-treated POMC-G12D mice.

### POMC-G12/13 KO mice show the expected deficits in Gα_12/13_ expression

We also carried out metabolic studies with mice that lacked Gα_12_ throughout the body and Gα_13_ selectively in POMC neurons (abbreviated name: POMC-G12/13 KO mice; genetic background: C57BL/6). Specifically, we crossed POMC-Cre mice with *Gna12−/− Gna13 fl/fl* mice (*78*). The resulting Cre-positive offspring was then backcrossed to *Gna12−/− Gna13 fl/fl* mice to obtain POMC-Cre *Gna12−/− Gna13 fl/fl* mice (POMC-G12/13 KO mice) and control littermates lacking the Cre transgene (*Gna12−/− Gna13 fl/fl* mice).

Western blotting studies showed that Gα_12_ was not detectable in lysates from the mediobasal hypothalamus or whole brain of *Gna12*−/− *Gna13 fl/fl* mice (fig. S14). In contrast, Gα_12_ could be easily detected in whole brain lysates prepared from wild-type mice (fig. S14). To confirm that Gα_13_ was absent in POMC neurons of POMC G12/13 KO mice, we prepared hypothalamic sections from POMC G12/13 KO mice and control littermates lacking the POMC-Cre transgene that had been injected with an AAV-DIO-mCherry virus into the ARC. By using a Gα_13_-specific antibody, we observed strong colocalization of Gα_13_ with mCherry, a marker for POMC neurons, in the control mice (fig. S15). In contrast, Gα_13_ staining was almost completely absent in POMC neurons of POMC G12/13 KO mice (fig. S15), indicative of greatly reduced Gα_13_ expression in POMC neurons of this mutant mouse strain.

### G12D activation causes improved glucose tolerance in a G_12/13_-dependent fashion

POMC-G12/13 KO mice and control littermates lacking the Cre transgene did not differ in body weight and glucose tolerance (fig. S16). Likewise, additional metabolic studies did not reveal any significant differences in blood glucose and plasma hormone levels between the two groups of mice, most likely due to the ability of other signaling pathways to compensate for the lack of G_12/13_ signaling in POMC neurons.

As discussed throughout the manuscript, the major phenotype displayed by DCZ-treated POMC-G12D mice was a marked improvement in glucose tolerance observed with both lean and obese mice. To confirm that this phenotype was caused by enhanced G_12/13_ signaling in the ARC POMC neurons, we carried out glucose tolerance tests with two strains of mice generated via injection of a G12D-encoding adeno-associated virus (AAV; AAV-DIO-G12D) into the ARC of POMC-Cre mice.

POMC-ARC-G12D mice express Cre and G12D in POMC neurons of the ARC, while POMC-G12/13 KO-ARC-G12D mice express Cre and G12D in ARC POMC neurons deficient in Gα_12_ and Gα_13_. Thus, these two mouse strains differ only by the G12/13 KO mutations. Colocalization studies showed that 46.1 ± 15.3% of ARC POMC neurons expressed the G12D receptor (fig. S17A; POMC-ARC-G12D mice) (see Materials and Methods for details).

When maintained on DCZ drinking water, POMC-ARC-G12D mice showed a marked increase in glucose tolerance (fig. S17B), as expected. In contrast, DCZ treatment failed to improve glucose tolerance in POMC-G12/13 KO-ARC-G12D mice (fig. S17C), clearly indicating that G12D-mediated activation of G_12/13_ signaling in ARC POMC neurons is responsible for the improvement in glucose tolerance caused by G12D activation.

### G12D activation of ARC POMC neurons triggers c-Fos and RhoA-GTP expression

We performed additional experiments to explore how G12D-mediated activation of G_12/13_ signaling affects the signaling properties of ARC POMC neurons. Specifically, we injected mice with DCZ (100 μg/kg, ip) 1 hour before the collection of brain tissue. DCZ treatment of POMC-ARC-G12D mice resulted in a strong c-Fos and RhoA-GTP signal in G12D+ neurons (figs. S18 and S19, respectively). Colocalization studies indicated that 33.0 ± 8.4 and 39.4 ± 5.3% of G12D+ neurons displayed prominent c-Fos and RhoA-GTP staining, respectively. RhoA-GTP (guanosine 5′-triphosphate), the active form of RhoA, represents a major downstream effector of G_12/13_ signaling (*18*).

Under the same experimental conditions, we observed little or no c-Fos and RhoA-GTP staining in mice expressing G12D in ARC POMC neurons of POMC-G12/13 KO mice or in DCZ-treated control mice that did not express G12D (POMC-ARC-mCherry mice) (figs. S18 and S19, respectively). POMC-ARC-G12D mice maintained on DCZ water for 3 days showed a pattern of RhoA-GTP formation in ARC POMC neurons that was similar to that observed after acute DCZ treatment of POMC-ARC-G12D mice (fig. S20). Under these experimental conditions, RhoA-GTP staining could be detected in 36.3 ± 4.5% of G12D+ neurons. These data indicate that activation of the G12D designer receptor expressed by ARC POMC neurons stimulates G_12/13_ signaling in these neurons in vivo, thus promoting RhoA signaling and neuronal activation.

### The anorectic and antidiabetic effects of lorcaserin require G_12_ signaling

Our final goal was to target an endogenous GPCR expressed by POMC neurons that can couple to G_12/13_, besides other functional classes of G proteins. In this study, we focused on the serotonin 5-HT_2C_ receptor subtype, which is considered a useful therapeutic target for various pathophysiological conditions including obesity and type 2 diabetes (*79*, *80*). Previous work has demonstrated that lorcaserin and related 5-HT_2C_ receptor agonists exert their anorectic and antidiabetic effects by activating 5-HT_2C_ receptors expressed by POMC neurons (*81*–*86*).

Agonist activation of 5-HT_2C_ receptors is known to trigger the activation of G proteins of the G_q_ family (*87*, *88*). However, recent in vitro studies using cell-based functional assays demonstrated that activated 5-HT_2C_ receptors can also interact with other functional classes of G proteins including G_i/o_ and G_12/13_ (*20*, *22*). Prompted by this recent work, we explored the ability of 5-HT_2C_ receptors expressed by POMC neurons to couple to G_12/13_ and the potential contribution of this interaction to the metabolic effects caused by stimulating central 5-HT_2C_ receptors.

**Fig. 9.**
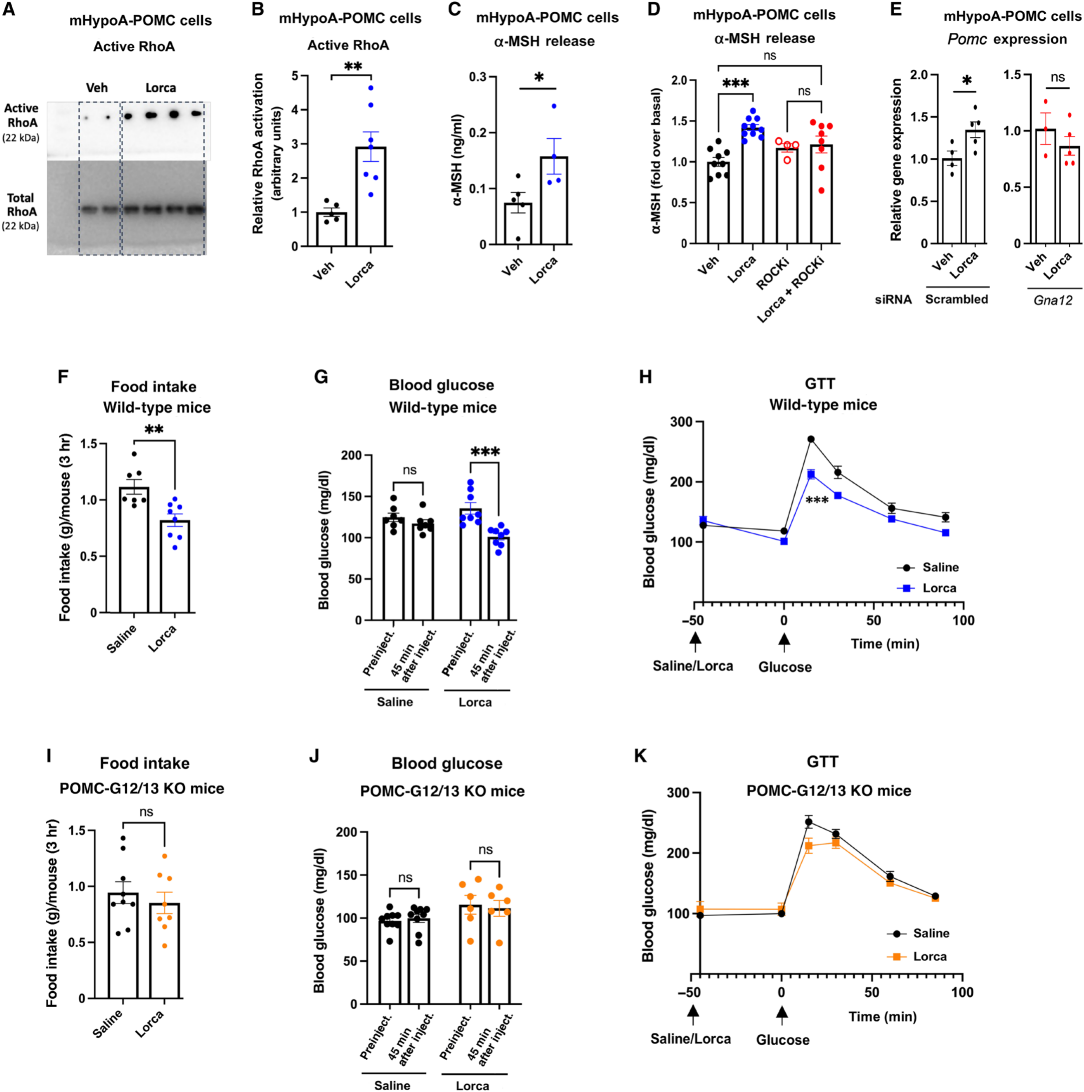
Lorcaserin exerts its beneficial metabolic effects via stimulation of G_12/13_ signaling in POMC neurons. All in vivo studies were carried out with male mice consuming regular chow. (**A**) Western blotting studies carried out with lorcaserin (Lorca)–treated mHypoA-POMC cells. Note that Lorca (1 μM; incubation time: 15 min) stimulated the formation of active RhoA. (**B**) Quantification of the Western blotting data shown in (A) (*n* = 5 to 7). (**C**) Lorca treatment (1 μM; incubation time: 60 min) of mHypoA-POMC cells stimulates α-MSH secretion (*n* = 4 or 5). (**D**) Lorca-induced stimulation of α-MSH secretion is abolished after 30-min pretreatment of cells with a selective ROCKi (Y27632, 10 μM) (*n* = 4 to 10). (**E**) Lorca treatment (1 μM; incubation time: 3 hours) increases *Pomc* gene expression, as determined via qRT-PCR. This response was abolished after treatment of cells with *Gna12* siRNA (*n* = 3 to 5). (**F** to **H**) Measurements of acute food intake for 3 hours (initiated at 6 p.m.) (F), fasted blood glucose levels (G), and GTT (H) in wild-type mice following a single injection of Lorca (10 mg/kg, ip) or saline (*n* = 7 or 8). (**I** to **K**) Measurements of acute food intake for 3 hours (initiated at 6 p.m.) (I), fasted blood glucose levels (J), and GTT (K) in POMC-G12/13 KO mice after a single injection of Lorca (10 mg/kg, ip) or saline (*n* = 6 to 9). Blood glucose and GTT measurements were performed with mice that had been fasted for 6 to 8 hours. Results shown in (F) to (K) were obtained with 12- to 14-week-old mice. Data are presented as the means ± SEM. *P < 0.05, **P < 0.01, and ***P < 0.001, as compared with the corresponding control group [unpaired *t* test (B), (C), (E), (F), and (I); two-way ANOVA, followed by multiple comparisons test (D), (G), and (J); two-way repeated measures ANOVA, followed by Šídák’s multiple comparisons test (H) and (K)].

Initially, we carried out studies with mHypoA-POMC cells to investigate whether lorcaserin was able to activate G_12_-type G proteins in a functional assay. Receptor-mediated activation of G_12/13_ signaling leads to the activation of various subtypes of Rho guanine nucleotide exchange factors, resulting in the formation of the active form of RhoA (RhoA-GTP) (*18*). Western blotting studies showed that incubation of mHypoA-POMC cells with lorcaserin (1 μM) for 15 min led to a robust increase (approximately threefold) in the formation of active RhoA (Fig. 9, A and B), suggesting that lorcaserin-induced activation of 5-HT_2C_ receptors stimulates G_12/13_ signaling in this cell line.

Lorcaserin (1 μM) also enhanced the release of α-MSH from mHypoA-POMC cells by approximately twofold (Fig. 9C). Because the activation of G_12/13_ signaling stimulates Rho kinase (ROCK) activity with high efficacy (*18*), we speculated that the lorcaserin-induced increase in α-MSH release required the activation of ROCK. In agreement with this hypothesis, lorcaserin-induced α-MSH secretion was abolished after pretreatment of mHypoA-POMC cells with a selective ROCK inhibitor (ROCKi; Y27632, 10 μM) (Fig. 9D).

A previous study (*89*) showed that 5-HT_2C_ receptor activation was associated with increased transcript levels of genes coding for hypothalamic peptides endowed with anorectic activity including *Pomc*. In agreement with this finding, lorcaserin treatment (1 μM) of mHypoA-POMC cells led to an increase in *Pomc* mRNA levels (Fig. 9E). Because 5-HT_2C_ receptors couple to G_12_ with considerably greater efficacy than to G_13_ (*20*), we treated mHypoA-POMC cells with *Gna12* siRNA. Following siRNA-mediated knockdown of *Gna12* expression, lorcaserin treatment had no significant effect on *Pomc* mRNA levels (Fig. 9E).

Prompted by these in vitro findings, we tested the hypothesis that the in vivo beneficial metabolic effects of lorcaserin required the activation of G proteins of the G_12_ family. We first demonstrated that lorcaserin treatment (10 mg/kg, ip) of wild-type C57BL/6 mice resulted in a reduction in food intake, decreased blood glucose levels, and greatly improved glucose tolerance (Fig. 9, F to H). Notably, these beneficial metabolic effects of lorcaserin were abolished in POMC-G12/13 KO mice (Fig. 9, I to K). These data convincingly demonstrate that the beneficial metabolic actions of lorcaserin depend on the activation of G_12/13_ signaling in POMC neurons.

### G protein coupling profiles after agonist activation of 5-HT_2C_ receptors in vitro

To explore which G protein families are activated after the binding of lorcaserin to 5-HT_2C_ receptors, we carried out NanoBiT-G protein dissociation assays using 5-HT_2C_ receptor–expressing human embryonic kidney (HEK) 293 cells that coexpressed NanoBiT sensors for G_12_, G_13_, G_q_, G_i1_, or G_s_ (fig. S21) (*22*). HEK293 cells that did not express the 5-HT_2C_ receptor (mock cells) were used for control purposes. We found that lorcaserin promoted signaling via G_12_, G_13_, G_q_, and G_i1_ in a concentration-dependent manner (G_s_ signaling was barely detectable). Under the chosen experimental conditions, lorcaserin activated G_q_ signaling with greater efficacy than G_12/13_ signaling (fig. S21). Treatment of 5-HT_2C_ receptor–expressing HEK293 cells with serotonin, the endogenous 5-HT_2C_ receptor agonist, resulted in a G protein activation profile that was very similar to that observed with lorcaserin (fig. S21).

## DISCUSSION

Although G proteins of the G_12_ family (G_12_/G_13_) are widely expressed throughout the brain (*17*), their potential roles in regulating key functions of the central nervous system remain unexplored. In the present study, we used a chemogenetic approach, combined with the use of loss-of-function mouse models, to explore the roles of G_12/13_ signaling in POMC neurons. This class of neurons is of preeminent importance for the regulation of energy and glucose homeostasis, including the autonomic control of peripheral metabolic tissues (*9*–*11*).

**Fig. 10.**
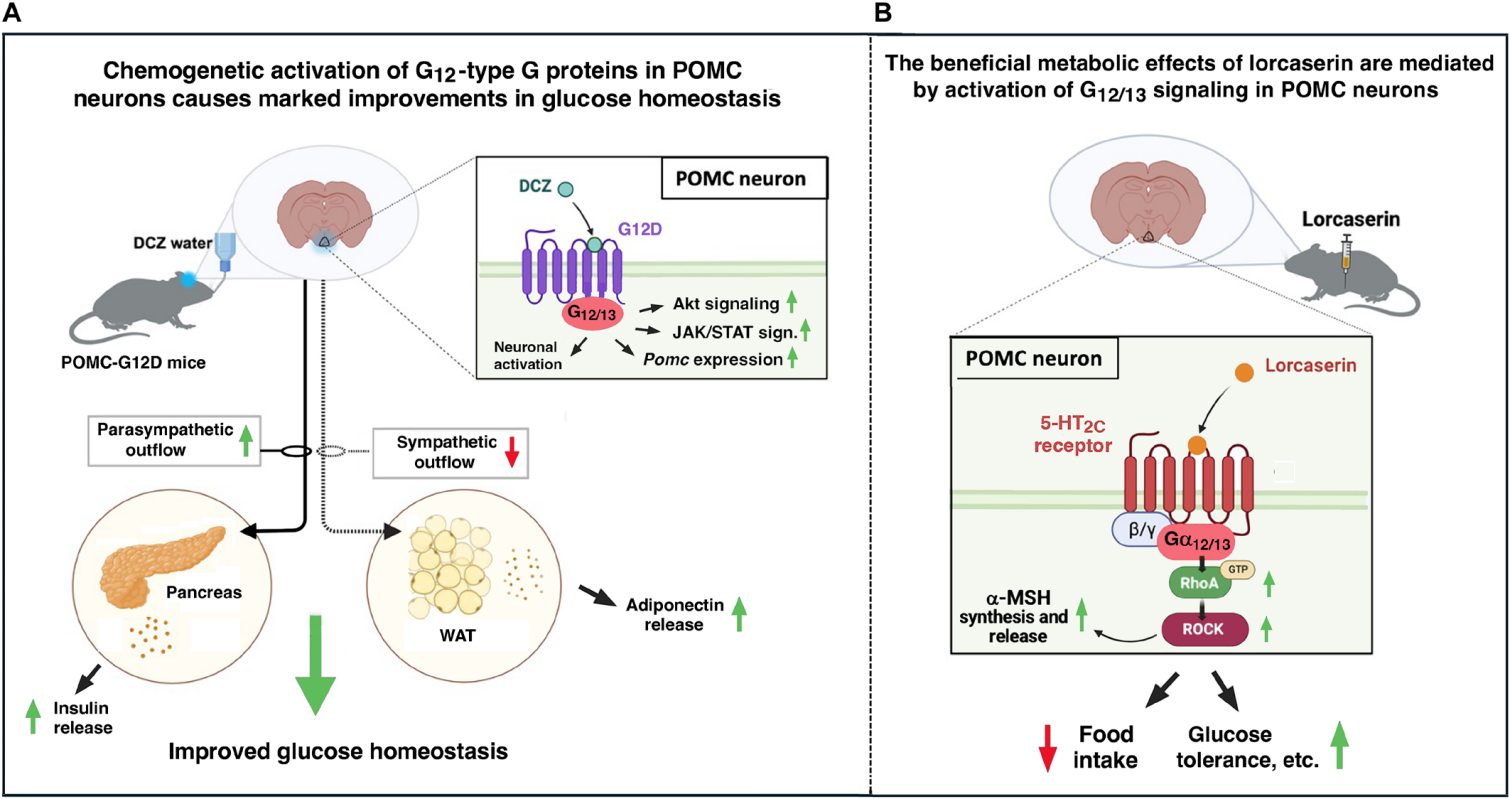
Scheme highlighting the major metabolic effects triggered by activation of G_12_-type proteins in POMC neurons. (**A**) Summary of mechanisms through which activation of G_12/13_ signaling in POMC neurons improves glucose homoeostasis. (**B**) Key role of G_12/13_ signaling in POMC neurons in mediating the beneficial metabolic effects of Lorca.

Using a chemogenetic strategy, we generated a mouse strain (POMC-G12D) that allowed us to selectively stimulate G_12/13_ signaling in POMC neurons in a ligand (DCZ or CNO)–dependent fashion. In parallel, we analyzed mice harboring inactivation mutations in the genes encoding Gα_12_ and Gα_13_ (*Gna12* and *Gna13*, respectively) selectively in POMC neurons. Systematic metabolic analysis of these newly generated mouse strains yielded several unexpected results. We found that chronic, pharmacological activation of G_12/13_ signaling in POMC neurons resulted in marked improvements in glucose tolerance and several other metabolic parameters in both male and female mice, while body weight remained unaltered (summarized in Fig. 10A). Moreover, G12D-mediated stimulation of G_12/13_ signaling in POMC neurons strongly enhanced glucose-stimulated insulin secretion and resulted in elevated plasma adiponectin levels. Most notably, genetic disruption of G_12/13_ signaling in POMC neurons virtually abolished the ability of lorcaserin, an appetite-suppressant 5-HT_2C_ receptor agonist, to reduce food intake and improve glucose homeostasis (summarized in Fig. 10B).

We demonstrated that activation of G_12/13_ signaling in ARC POMC neurons resulted in a delayed hypophagic effect (Fig. 1E). In agreement with this observation, previous studies have shown that modulation of the activity of ARC POMC neurons leads to delayed effects on food intake [reviewed in (*90*)]. For example, chemogenetic (*34*) or optogenetic (*35*) activation of ARC POMC neurons causes late-onset hypophagic responses. A more recent study (*91*) also reported that chemogenetic stimulation of ARC POMC neurons reduces appetite with a major delay (3 days). The molecular and cellular mechanisms underlying these delayed anorectic effects remain unclear at present but are probably related to changes in synaptic organization (*90*).

POMC-G12D mice consuming DCZ drinking water showed long-lasting improvements in glucose homeostasis including improved glucose tolerance. These beneficial metabolic effects were observed with POMC-G12D mice maintained on either regular chow or an obesogenic HFD. This latter observation suggests that pharmacological strategies that can promote G_12/13_ signaling in POMC neurons may prove useful to restore euglycemia under pathophysiological conditions characterized by impaired glucose tolerance.

Following glucose administration, obese POMC-G12D mice consuming DCZ water showed significant increases in plasma insulin and plasma PP levels. Previous studies have shown that elevated plasma PP levels are caused by enhanced vagal (parasympathetic) flow to the pancreas (PP is released from PP cells of pancreatic islets) (*42*, *49*, *50*). Because increased vagal activity represents a major stimulus for insulin secretion from the endocrine pancreas (*41*–*43*), this observation can explain why chronic DCZ treatment led to elevated plasma insulin levels and improved glucose tolerance in POMC-G12D mice.

To further corroborate the concept that chronic activation of G_12/13_ signaling in POMC neurons stimulates vagal outflow, we acutely treated POMC-G12D mice consuming DCZ drinking water with NMA, a peripherally acting muscarinic acetylcholine receptor antagonist that blocks the peripheral effects of vagal activation (*44*, *45*). We found that NMA treatment virtually abolished the improvement in glucose tolerance displayed by POMC-G12D mice consuming DCZ water. Together, these observations strongly suggest that sustained pharmacological activation of G_12/13_ signaling in POMC neurons improves glucose homeostasis by promoting vagal output to the pancreas (Fig. 10A). In agreement with our findings, a recent study (*92*) demonstrated that mice with reduced excitatory glutamatergic input into POMC progenitor neurons exhibit impaired glucose tolerance resulting from reduced vagus nerve activity and impaired insulin release.

Consistent with our observations, previous work has established that certain hypothalamic neurocircuits, including pathways regulated by the activity of POMC neurons, increase autonomic outflow to various peripheral tissues including different adipose depots and pancreatic islets (*93*–*96*). Retrograde-tracing studies demonstrated that neuronal populations in the ARC, including POMC neurons, and other regions of hypothalamus communicate with distinct groups of neurons in the brain stem (nucleus of the solitary tract and dorsal motor nucleus of the vagus) (*93*–*95*). It has also been shown that specific brain stem neurons innervate the endocrine pancreas via the vagus nerve (*94*). Notably, a recent study (*95*) demonstrated that pancreatic islets are innervated by efferent circuits emanating from the hypothalamus, in particular from the ARC, resulting in enhanced vagal outflow.

Another major phenotype displayed by DCZ-treated POMC-G12D mice (both males and females) was a significant elevation of plasma adiponectin levels. In addition, we found that *Th* mRNA levels and NE content were markedly reduced in the WAT of DCZ-treated POMC-G12D mice, indicative of reduced sympathetic activity in WAT. Previous studies (*56*, *57*) have shown that decreased sympathetic flow to mouse WAT leads to increased adiponectin secretion. Together, these observations strongly suggest that sustained pharmacological activation of G_12/13_ signaling in POMC neurons reduces sympathetic flow to WAT, which in turn leads to increased *Adipoq* expression and elevated plasma adiponectin levels (Fig. 10).

Somewhat unexpectedly, the decrease in sympathetic outflow displayed by DCZ-treated POMC-G12D mice did not cause significant changes in fat mass and body weight. In agreement with this observation, it has been reported that changes in sympathetic tone can lead to altered plasma adiponectin levels without affecting body weight or fat mass. For instance, cold exposure of mice for 24 hours, which elevates sympathetic tone, results in reduced circulating adiponectin levels without affecting body weight, WAT mass, and adipocyte size (*57*). Moreover, certain pathological conditions characterized by elevated sympathetic tone are associated with changes in plasma adiponectin levels without alterations in body weight, fat mass, and fat distribution (*97*, *98*). The cellular and molecular mechanisms underlying these selective effects of altered sympathetic tone on circulating adiponectin levels remain unknown at present. One possibility is that adiponectin expression by adipocytes is particularly sensitive to changes in the activity of the sympathetic nervous system.

We also noted that sympathetic tone was not significantly altered in BAT following activation of G12D signaling in POMC neurons (Fig. 5B). Because BAT plays a key role in regulating energy expenditure in mice, this finding may contribute to the observation that body weight remained unchanged in DCZ-treated POMC-G12D mice.

Many of the important metabolic actions of leptin are mediated by activation of leptin receptors expressed by POMC neurons (*38*, *99*, *100*). We demonstrated that G12D-mediated stimulation of G_12/13_ signaling activated similar downstream signaling cascades as leptin. Like leptin, G12D-induced G_12/13_ signaling led to a marked stimulation of JAK2-STAT3 and Akt signaling. Moreover, leptin treatment of POMC-G12D mice consuming DCZ drinking water resulted in more robust decreases in body weight and food intake, as compared with leptin-treated control littermates lacking the G12D receptor. These observations clearly indicate that pharmacological activation of G_12/13_ signaling in POMC neurons enhances the beneficial metabolic effects of leptin.

Leptin receptor signaling in POMC neurons is required for proper *Pomc* gene expression (*29*). In agreement with the observation that G_12/13_ signaling in POMC neurons stimulates similar signaling pathways to leptin (see the previous paragraph), DCZ treatment led to a significant increase in *Pomc* expression in the ARC of POMC-G12D mice. We obtained similar data with G12D-expressing mHypoA-POMC cells. It is likely that the G_12/13_-mediated increase in *Pomc* expression contributes to the beneficial metabolic effects displayed by DCZ-treated POMC-G12D mice.

A previous study (*82*) convincingly demonstrated that the metabolic profile of POMC-Cre mice did not differ significantly from that of wild-type littermates. This study included measurements of food intake, body weight, blood glucose and plasma insulin and glucagon levels, energy expenditure, fuel usage, locomotor activity, body composition, and 5-HT_2C_ receptor agonist–mediated inhibition of food intake (*82*). In agreement with this previous report, we found that POMC-G12/13 KO mice (genotype: *Pomc-Cre Gna12−/− Gna13 fl/fl* mice) and their control littermates lacking the *Pomc-Cre* transgene (*Gna12−/− Gna13 fl/fl* mice) did not differ in body weight and glucose tolerance (fig. S16) and several other metabolic parameters. For this reason, we consider it unlikely that potential developmental changes caused by the *Pomc-Cre* transgene interfere with the major conclusions drawn from our experimental data.

In contrast to the robust metabolic phenotypes displayed by DCZ-treated POMC-G12D mice, POMC-G12/13 KO mice (mice lacking Gα_12_ throughout the body and Gα_13_ selectively in POMC neurons) did not show any obvious metabolic changes. The most likely explanation for this observation is that other signaling pathways can function to maintain proper glucose and energy homeostasis in the absence of G_12/13_ signaling in POMC neurons. There are many examples in the literature that are consistent with this conclusion. For example, while Agrp is a highly efficacious appetite-inducing neuropeptide, Agrp KO mice show no obvious metabolic phenotypes (*101*). Moreover, while GLP-1 receptor agonists have emerged as powerful appetite-suppressing drugs, GLP-1 receptor KO mice do not develop obesity (*102*).

Our ultimate goal was to target a G_12/13_-linked GPCR that is endogenously expressed by POMC neurons. Recent work has identified dozens of GPCRs that can interact with G_12/13_ with moderate to high efficacy (*20*–*22*). In the present study, we focused on the 5-HT_2C_ receptor subtype that couples to both G_12_ and G_13_ but shows a clear preference for G_12_ (*20*). Published data indicate that agonist-stimulated 5-HT_2C_ receptors can also couple to G proteins of the G_q_ and G_i_ families (also see fig. S21) (*87*, *103*). The 5-HT_2C_ receptor is highly expressed in POMC neurons and is the target of lorcaserin, an antiobesity drug (*82*, *85*, *86*). Besides its appetite-suppressant effect, lorcaserin treatment also leads to improved glucose homeostasis, independent of changes in body weight (*81*, *82*, *85*, *86*, *104*, *105*). Previous work has shown that the lorcaserin-mediated improvements in glucose homeostasis are mediated by 5-HT_2C_ receptors expressed by POMC neurons (*82*). Moreover, a recent study identified several rare variants of the *5HTR2C* gene in individuals with severe obesity, further highlighting the metabolic importance of this receptor subtype (*106*).

Lorcaserin’s anorectic and beneficial effects on glucose homeostasis were either completely absent or greatly diminished in mice lacking Gα_12/13_ signaling in POMC neurons (POMC-G12/13 KO mice). In vitro studies demonstrated that lorcaserin increased *Pomc* gene expression and enhanced α-MSH release in cultured mHypoA-POMC cells. We also showed that these lorcaserin effects required the presence of Gα_12_ and the activation of ROCK, a major downstream target of G_12/13_ signaling (summarized in Fig. 10B) (*18*).

Together, these data support the concept that the beneficial metabolic actions of lorcaserin depend on the ability of 5-HT_2C_ receptors to stimulate G_12/13_ signaling in POMC neurons. Clearly, this finding is of considerable translational relevance. It is conceivable that agents capable of selectively activating G_12/13_ signaling in POMC neurons show increased clinical efficacy and an improved side effect profile, as compared with lorcaserin that can also activate other functional classes of G proteins (*88*, *107*, *108*).

A recent study demonstrated that lorcaserin treatment can improve synaptic plasticity and memory in a mouse model of Alzheimer’s disease (*109*). It should be of considerable interest to explore the possibility that this lorcaserin effect also requires the activity of G proteins of the G_12_ family.

In conclusion, we found that pharmacological activation of G_12/13_ signaling in POMC neurons has strong beneficial effects on several key metabolic functions (summarized in Fig. 10). We identified a mechanism by which 5-HT_2C_ receptors expressed by POMC neurons regulate appetite and glucose homeostasis. The outcome of the present study is likely to stimulate the development of strategies aimed at enhancing G_12/13_ signaling in POMC neurons for the treatment of obesity, type 2 diabetes, and related metabolic disorders.

### Limitations of the study

To further corroborate the potential therapeutic relevance of the data presented in this study, it remains to be demonstrated that a similar G_12/13_ signaling pathway is also operative in human POMC neurons. It should also be of interest to investigate whether G_12/13_ signaling contributes to the effects of other appetite-suppressant drugs known to act on POMC neurons. Last, future studies need to explore which specific GPCRs capable of coupling to G_12_-type G proteins are enriched in human POMC neurons.

## MATERIALS AND METHODS

### Study approval

All animal studies were performed according to the National Institutes of Health Guide for the Care and Use of Laboratory Animals and approved by the Institutional Animal Care and Use Committees of the University of Texas and the National Institute of Diabetes and Digestive and Kidney Diseases [NIDDK; National Institutes of Health (NIH), Bethesda, MD].

### Drugs, reagents, commercial kits, and antibodies

The sources of all drugs, reagents, antibodies, and mouse strains are listed in table S1.

### Mouse models

We crossed *Rosa26-LSL-G12D-IRES-GFP* mice (short name: LSL-G12D mice) (*24*) with POMC-Cre mice (*29*) to generate POMC-Cre^+^ LSL-G12D mice (short name: POMC-G12D mice). LSL-G12D mice without the Cre transgene served as control mice. The POMC-Cre mice were originally obtained from the Jackson Laboratory (stock no. 005965) and then backcrossed for eight generations onto the C57BL/6Tac background in our animal facility. POMC neuron–specific G12/13 KO mice (POMC-G12/13 KO mice) were generated by crossing POMC-Cre mice with *Gna12−/− Gna13 fl/fl* mice (*78*). *Gna12−/− Gna13 fl/fl* mice lacking the Cre transgene or age-matched POMC-Cre mice served as control mice in studies where POMC-G12/13 KO mice were analyzed. In a subset of experiments, wild-type C57BL/6 mice were used (C57BL/6NTac mice from Taconic).

### Mouse maintenance and diet

All experiments were carried out with adult mice that were at least 8 weeks old. For most experiments, mice were maintained at room temperature (23°C), consuming standard rodent chow (LabDiet, cat. no. 5018; energy density, 3.05 kcal/g). The mice had free access to water and food and were kept on a 12-hour light, 12-hour dark cycle (lights off at 6 p.m.). In a subset of experiments, 24-week-old mice were switched to an HFD (F3282, 50% kcal fat; energy density, 5.5 kcal/g; Bioserv). Mice consumed the HFD for up to 14 weeks.

### AAV-hSyn-DIO viruses and stereotaxic surgery

An AAV (serotype 8) coding for G12D (AAV8.hSyn.DIO.HA-G12D-mCherry; short name: AAV-DIO-G12D) was custom-made by the Penn Vector Core (University of Pennsylvania, PA). For control purposes, we also used a pharmacologically inert AAV (serotype 8) coding for mCherry (AAV8.hSyn.DIO.mCherry; short name: AAV-DIO-mCherry) (Addgene). Adult male mice were anesthetized with isoflurane (5% induction and 1 to 2% for maintenance) and placed in a stereotaxic apparatus (David Kopf). Surgery was performed as described previously (*110*). The AAV-DIO-G12D or AAV-DIO-mCherry viruses were injected into the ARC of POMC-Cre mice (virus concentrations used for injections: 2 × 10^7^ infectious units/ml). All virus injections were performed bilaterally (200 nl per site). The coordinates for ARC injections were as follows: anterior-posterior, −1.46 mm (from bregma); lateral (from midline), ±0.3 mm; dorsal-ventral, −5.80 mm). Postoperative analgesia was provided by a single dose of sustained-release meloxicam ER (1.5 mg/kg, sc) (ZooPharm). The injected mice were allowed to recover for 1 to 2 weeks before metabolic testing.

### Immunohistochemistry and imaging studies

POMC-G12D mice and AAV-injected mice were anesthetized and transcardially perfused with 4% paraformaldehyde in 0.1 M phosphate buffer fixative (pH 7.4). Subsequently, brains were collected, postfixed in 4% paraformaldehyde, and cryoprotected by transferring to 20% sucrose in phosphate-buffered saline (PBS). Hypothalamic slices (30 μm thick) were prepared by using the Leica vibratome VT1000S or the Leica Cryostat CM3050S. Sections were incubated overnight at 4°C with different combinations of primary antibodies diluted in PBS supplemented with 1% bovine serum albumin (BSA) and 0.1% Triton X-100. Primary antibodies (dilution: 1:1000) against mCherry, HA-tag, POMC, c-Fos, p-STAT3, RhoA-GTP, and Gα_13_ were used to detect G12D in POMC neurons. Slices were then washed three times with PBS containing 0.1% Triton X-100 and incubated with fluorophore-conjugated secondary antibodies (Alexa Fluor 488 and Alexa Fluor 594; 1:1000 dilution) for 2 hours at room temperature. Last, slices were rinsed twice with wash buffer (PBS with 0.5% Triton X-100) and then mounted using Vectashield to preserve fluorescence (Vector Labs). Fluorescence images were taken with a Keyence BZ-9000 automated fluorescence microscope.

Immunohistochemistry staining for p-STAT3, c-Fos, and RhoA-GTP was also performed with mice that had been injected with DCZ (100 μg/kg, ip) before an overnight fast. Brain tissue was collected 1 hour after DCZ treatment. A subset of mice was maintained on DCZ drinking water (10 μg/ml) for 3 days, followed by immunohistochemistry staining of hypothalamic slices for p-STAT3 and RhoA-GTP. Brain tissue was collected after an overnight fast.

### Use of DCZ drinking water

In several experiments, mice maintained on regular chow were provided with drinking water supplemented with DCZ (10 μg/ml) for 2 to 4 weeks. Food intake was measured during the first week of DCZ treatment. Other metabolic studies, including plasma hormone measurements, were performed 2 to 4 weeks after initiation of DCZ treatment, unless stated otherwise. A separate cohort of mice was maintained on both HFD and DCZ water for up to 4 months. Bottles containing DCZ water were topped off every week. Bottles were replaced every 2 weeks with bottles containing freshly prepared DCZC water (10 μg/ml).

### Food intake measurements

Adult mice consuming regular water were acclimatized to single housing for at least 1 week before initiating food intake measurements. For acute food intake studies, CNO (5 mg/kg, ip) or lorcaserin (10 mg/kg, ip) were injected between 5 and 6 p.m. before the beginning of the dark cycle. Food intake was measured at defined postinjection time points. For chronic food intake studies, DCZ was added to the drinking water (10 μg/ml), and food intake was measured daily over the course of 1 week.

### In vivo metabolic tests

Intraperitoneal GTTs were performed after a 6- to 8-hour fast at around 5 p.m. Insulin tolerance tests were carried out after a 3- to 4-hour fast at around 5 p.m. Mice received either intraperitoneal glucose (1 or 1.5 g/kg) or insulin (1 U/kg), and blood glucose measurements were taken using tail vein blood at defined postinjection time points. Drugs were either co-injected with glucose (CNO, 5 mg/kg, ip) or injected 45 min before glucose treatment (lorcaserin, 10 mg/kg, ip).

### Indirect calorimetry and energy expenditure measurements

Energy expenditure measurements were carried out with mice at 22°C using an Oxymax/CLAMS monitoring system (Columbus Instruments). Mice consuming regular chow were acclimatized in metabolic chambers for 3 days and then treated with a single injection of CNO (5 mg/kg, ip) (acute studies). For chronic studies, mice were maintained on DCZ drinking water (10 μg/ml). Food intake, water intake, O_2_ consumption, CO_2_ production, and ambulatory activity were measured at 5-min intervals. After a single CNO injection, mice were monitored for 3 days. Mice maintained on DCZ drinking water were monitored for 7 days.

### Body composition analysis

The lean/fat mass composition of mutant and control mice was measured using the 3-in-1 Echo MRI Analyzer (Echo Medical System).

### Treatment of mice with leptin

Before the start of the study, adult POMC-G12D mice were maintained on DCZ drinking water (10 μg/ml) for 3 weeks to chronically stimulate G12D signaling in POMC neurons. Subsequently, singly housed mice received intraperitoneal saline injections (twice daily) for 3 days. On day 4, mice were injected with intraperitoneal leptin twice a day (2.5 mg/kg per injection; injection times: 10 a.m. and 5:30 p.m.). Food intake and body weight were measured before the start of leptin injections and daily during the 3-day leptin treatment period. Body weight and food intake measurements were carried out 1 to 2 days after the termination of leptin injections.

### Plasma hormone measurements

To determine plasma hormone levels, 30 to 40 μl of blood was collected from the tail vein of mice using heparin-coated microcapillary tubes. Plasma was then obtained by centrifugation at 4°C for 10 min at 10,000*g*. ELISA (enzyme-linked immunosorbent assay) kits were used to measure plasma levels of insulin, adiponectin, PP, leptin, and corticosterone by following the manufacturers’ instructions.

### Propranolol study

Adult mice consuming regular chow were maintained on drinking water containing both DCZ (10 μg/ml) and propranolol (0.5 mg/ml) for a period of 2 weeks. Blood plasma was collected immediately before DCZ/propranolol administration and weekly after placing the mice on DCZ/propranolol drinking water. An intraperitoneal GTT was performed 2 weeks after initiating the DCZ/propranolol treatment protocol.

### NMA treatment

Adult mice consuming regular chow were maintained on DCZ drinking water for 3 weeks, followed by an intraperitoneal GTT. To explore whether the outcome of the GTT was affected by inhibiting the activity of peripheral parasympathetic nerves, mice were injected with two doses of NMA (5 mg/kg, ip). Dose 1 was given at 10 a.m., and dose 2 was administered at 5 p.m. (*44*, *45*). Forty-five minutes after the second NMA injection, mice received an intraperitoneal glucose bolus (1.5 g/kg), followed by the monitoring of blood glucose levels. Food was withdrawn after the first NMA injection.

### ADP 400 treatment

POMC-G12D mice and control littermates (males) consuming regular chow and DCZ drinking water for 2 to 3 weeks were subjected to an intraperitoneal GTT after pretreatment with ADP 400, an adiponectin receptor antagonist (*59*–*62*). Mice were injected with two doses of ADP 400 (1 mg/kg, sc; 1 and 7 hours before glucose administration) before treatment with intraperitoneal glucose (1.5 g/kg). Food was withdrawn after the first ADP 400 injection. Blood glucose levels were monitored using blood obtained from the tail vein at defined postinjection time points.

### WAT NE measurement

Frozen WAT depots (BAT, iWAT, and eWAT) were homogenized in 0.01 M HCl in the presence of 0.15 mM EDTA and 4 mM sodium metabisulfite. Tissue lysates were centrifuged twice at 12,000*g* for 10 min to remove debris. NE levels were quantified as per the manufacturer’s instructions (Rocky Mountain Diagnostics).

### DCZ drinking water stability test

An aqueous DCZ solution (10 μg/ml) was prepared and stored at room temperature (23°C) for 1, 2, or 4 weeks. After the indicated time periods, 10 μl of DCZ solution was injected into an Agilent 1200 Series System with a diode array detector and a 2.1 by 150 mm Zorbax 300SB-C18 5 μm column. High-resolution mass spectra were acquired in positive ion mode using an Agilent G6230BA Accurate Mass TOF with an electrospray ionization source. The flow rate was set to 0.4 ml/min with water containing 0.1% formic acid as solvent A and acetonitrile containing 0.1% formic acid as solvent B.

### mHypoA-POMC cell culture, transfection, and drug treatment

mHypoA-POMC/GFP-2 cells (short name: mHypoA-POMC cells) (Cedarlane) were cultured in six-well plates in Dulbecco’s modified Eagle’s medium (DMEM; 4.5 g glucose/liter) containing 10% fetal bovine serum (FBS). To achieve G12D expression, cells were incubated with an adenovirus coding for G12D (Ad-CMV-HA-G12D) (Vector Biolabs). For control purposes, cells were treated with an adenovirus coding for eGFP (enhanced green fluorescent protein; Ad-CMV-eGFP) (Vector Biolabs) (virus concentrations: 1 × 10^11^ to 1 × 10^12^ infectious units/ml). Media were changed 24 hours later, fresh DMEM (4.5 g glucose/liter) containing 10% FBS was added, and the cells were allowed to grow for another 48 hours in a 5% CO_2_ incubator at 37°C.

For Gα_12_ knockdown experiments, 2 × 10^5^ cells per well grown in six-well plates were treated with siRNA targeting *Gna12* [ON-TARGETplus Mouse *Gna12* (14673; Horizon Discovery)] using Lipofectamine RNAiMax reagent (Invitrogen) in Opti-MEM media. For control purposes, cells were treated with scrambled siRNA [ON-TARGETplus Non-targeting pool (Horizon Discovery)] in a similar fashion. Both siRNAs were used at a concentration of 50 nM. Media were changed 8 to 10 hours after siRNA treatment, and cells were allowed to grow for an additional 48 to 72 hours in DMEM (4.5 g glucose/liter) containing 10% FBS in a 5% CO_2_ incubator at 37°C. About 12 hours before the addition of drugs, the cells were washed with PBS, and the media were replaced with low-glucose DMEM (1 g/liter) without FBS. Cells were incubated with the following drugs: DCZ (50 nM), leptin (100 nM), insulin (100 nM), or a combination of different drugs. In addition, lorcaserin (1 μM) was added to the cells either alone or following a 30-min pretreatment of the ROCKi (Y27632, 10 mM). Incubation times ranged from 15 min for immunoblotting experiments to 30 to 60 min for the measurement of α-MSH secretion. For gene expression analysis, drug treatments were carried out for 3 hours. Cells were incubated with drugs in a 5% CO_2_ incubator at 37°C.

### RhoA activation assay

mHypoA-POMC cells were seeded into six-well plates at a density of 2 × 10^5^ cells per well. Cells were cultured in DMEM (4.5 g glucose/liter) containing 10% FBS for 24 hours at 37°C in a CO_2_ incubator. The cells were then washed with PBS, and the media were replaced with DMEM (4.5 g glucose/liter) without FBS. Protein (500 μg) was prepared from serum-starved cells after 15 min, and RhoA bead pull-down assays were performed as per the manufacturer’s instructions (Cytoskeleton).

### Gene expression analysis

Cell or tissue lysates were homogenized, and RNA was extracted using the Direct-zol RNA Microprep Kit (Zymo Research). cDNA was synthesized from 1 to 2 μg of RNA using the ZymoScript RT PreMix Kit (Zymo Research). qRT-PCR was performed on cDNA by using SYBR Green Master Mix (Bio-Rad) using standard conditions (*111*). Gene expression data were normalized relative to β-actin transcript levels by using the ΔΔ*C*_t_ method (*112*). Primers are listed in table S2.

### Immunoblotting

Cell or tissue lysates were homogenized in radioimmunoprecipitation assay lysis buffer (Thermo Fisher Scientific) supplemented with protease and phosphatase inhibitors (Roche). Protein concentrations were determined by using the bicinchoninic acid assay method (Thermo Fisher Scientific). Loading buffer was added to 20 μg of protein lysate, and the resulting mixture was subjected to denaturing conditions (75°C for 5 min or 37°C for 10 min). The denatured samples were then resolved using SDS–polyacrylamide gel electrophoresis gradient gels and transferred onto a nitrocellulose membrane by semidry transfer (Bio-Rad). The membrane was then blocked using tris-buffered saline (TBS) containing 5% BSA and 0.1% Tween 20 (TBS-T). Subsequently, the membrane was incubated overnight at 4°C in the presence of the primary antibody. The membrane was then washed three times with TBS-T and incubated at room temperature with a horseradish peroxidase–conjugated anti-rabbit or anti-mouse secondary antibody. After three additional washing steps, immunoreactive bands were visualized using chemiluminescence technology (Azure Biosystems).

### α-MSH measurement

mHypoA-POMC cells were seeded into six-well plates at a density of 2 × 10^5^ cells per well. The cells were cultured in DMEM (4.5 g glucose/liter) containing 10% FBS for 48 to 72 hours. The cells were then washed with PBS, and the media were replaced with low-glucose DMEM (1 g/liter) without FBS. About 12 hours later, different drugs were added [DCZ (50 nM), leptin (100 nM), or lorcaserin (1 μM)]. The cells were then incubated for 30 to 60 min in a 5% CO_2_ incubator at 37°C. Following drug treatment, cell culture media were collected and centrifuged at 1500*g* at 4°C to remove cell debris. Protein was precipitated from the supernatant using the ProteoExtract Protein Precipitation kit, as per the manufacturer’s protocol (Sigma-Aldrich). The precipitated protein (1 to 100 μg/ml) was dissolved in 50 μl of PBS. The amount of α-MSH present in the protein precipitate was determined by using an α-MSH ELISA kit (Phoenix Pharmaceuticals).

### Brain slice preparation for electrophysiological recordings

Brain slices were prepared from POMC-G12D mice and AAV-injected mice as previously described (*113*–*115*). Briefly, male mice were deeply anesthetized with an intraperitoneal injection of 7% chloral hydrate (10 μl/g) and transcardially perfused with a modified ice-cold artificial cerebrospinal fluid (aCSF) (described below). The mice were then decapitated, and the entire brain was removed and immediately submerged in ice-cold, carbogen-saturated (95% O_2_ and 5% CO_2_) aCSF (126 mM NaCl, 2.8 mM KCl, 1.2 mM MgCl_2_, 2.5 mM CaCl_2_, 1.25 mM NaH_2_PO_4_, 26 mM NaHCO_3_, and 5 mM glucose). Coronal sections (250 μm) from the entire brain including the hypothalamus were cut with a Leica VT1000S Vibratome and then incubated in oxygenated aCSF (32° to 34°C) for at least 1 hour before recording. The slices were bathed in oxygenated aCSF (32° to 34°C) at a flow rate of ∼2 ml/min. All electrophysiology recordings were performed at room temperature.

### Whole-cell recordings

The pipette solution for whole-cell recordings was as follows: 125 mM K-gluconate, 2 to 10 mM KCl, 10 mM Hepes, 5 to 10 mM EGTA, 1 mM CaCl_2_, 1 mM MgCl_2_, 1 to 2 mM MgATP, and 0.03 mM Alexa Fluor 350 hydrazide dye (pH 7.3). Electrophysiological recordings were performed similar to previous reports (*113*–*115*). Briefly, epifluorescence was used to target ARC POMC neurons that were identified by Cre-dependent mCherry fluorescence, at which time the light source was switched to infrared differential interference contrast imaging to obtain the whole-cell recording (Zeiss Axioskop FS2 Plus equipped with a fixed stage and a QuantEM:512SC electron-multiplying charge-coupled device camera). Electrophysiological signals were recorded using an Axopatch 700B amplifier (Molecular Devices), low-pass filtered at 2 to 5 kHz, and analyzed offline on a PC with a patch-clamp (pCLAMP) electrophysiology data acquisition and analysis program (Molecular Devices). Membrane potentials were determined from ARC POMC neurons expressing G12D in brain slices. Recording electrodes showed a resistance of 2.5 to 5 megohms when filled with the K-gluconate internal solution.

### Analysis of electrophysiological recordings

A change in membrane potential was required to be at least 2 mV in amplitude. Membrane potential values were not compensated to account for junction potential (−8 mV). All graphs and figures were generated using either GraphPad Prism 10.0 software (GraphPad Software Inc.) or CorelDraw X8 (Corel Corp.).

### NanoBiT-G protein dissociation assay

5-HT_2C_ receptor–stimulated G protein dissociation was measured by the NanoBiT-G protein dissociation assay (*22*) with modifications as described below. One day before transfection, HEK293A cells (Thermo Fisher Scientific, cat. no. R70507) were seeded into six-well culture plates at a density of 2 × 10^5^ cells/ml [medium: 2 ml of DMEM (Nissui) supplemented with 5% FBS (Gibco) and penicillin-streptomycin-glutamine; complete DMEM]. Cells were transfected with NanoBiT-G protein sensors composed of Gα (Gα_12_, Gα_13_, Gα_q_, Gα_i1_, or Gα_s_) containing the large fragment of NanoBiT luciferase (LgBiT) inserted into the helical domain, Gγ_2_ containing the C68S point mutation and the N-terminal small fragment of NanoBiT luciferase (SmBiT-Gγ_2_-CS), and untagged Gβ_1_. These three constructs were expressed either alone (NanoBiT-G_i1_), together with RIC8A (NanoBiT-G_12_, NanoBiT-G_13_, and NanoBiT-G_q_), or combined with RIC8B (NanoBiT-G_s_). The transfection solution was prepared by combining 6 μl (per well hereafter) of polyethyleneimine PEI MAX solution (1 mg/ml; Polysciences) with 200 μl of Opti-MEM (Thermo Fisher Scientific) containing plasmid DNA (200 ng) encoding the human 5-HT_2C_ receptor (or empty vector for mock transfections), 100 ng of Gα-LgBiT, 500 ng of Gβ_1_, 500 ng of SmBiT-Gγ_2_-CS, and 100 ng of RIC8 plasmid DNA. After a 1-day incubation period, the transfected cells were harvested with Dulbecco’s PBS containing 0.5 mM EDTA, centrifuged, and suspended in 2 ml of Hanks’ balanced salt solution containing 0.01% BSA (fatty acid-free grade; Serva) and 5 mM Hepes assay buffer (pH 7.4). The cell suspension was dispensed into a white 96-well plate (80 μl per well) and combined with 20 μl of 50 μM coelenterazine (Angene) diluted in assay buffer. After a 2-hour incubation at room temperature, background luminescence was determined using a luminescence microplate reader (SpectraMax L, Molecular Devices), and defined amounts of 5-HT_2C_ receptor agonists (serotonin or lorcaserin) (20 μl; 6× of final concentrations) were added. Luminescence signals were measured 5 to 10 min after the addition of the two agonists and normalized to the initial counts. The resulting values (fold change) were further normalized to those obtained with vehicle-treated samples and used to generate G protein dissociation-response curves. The G protein dissociation signals were fitted to a four-parameter sigmoidal concentration-response curve.

### Statistical analyses

Before determining statistical significance, tests for normality and homogeneity of variance were performed. When only two groups were compared, a two-tailed Student’s *t* test was performed (unpaired or paired, as appropriate). When more than two groups were compared, a one- or two-way analysis of variance (ANOVA) or a repeated measures ANOVA, followed by the proper post hoc tests, was carried out. A *P* value of < 0.05 was considered statistically significant. The statistical tests used for data analysis are indicated in the individual figure legends.
