# Supplementary material for: G proteins of the G12 family expressed by POMC neurons regulate key metabolic functions
Source: Sci Adv. 2025 Jul 11;11(28):eadu1670. doi: 10.1126/sciadv.adu1670 (PMC12248387; doi:10.1126/sciadv.adu1670)
Supplement: Supplementary file 1 — Figs. S1 to S21 Tables S1 and S2 [file sciadv.adu1670_sm.pdf]

Science Advances
AAAS

Supplementary Materials for

G proteins of the G_{12} family expressed by POMC neurons regulate key metabolic functions

Dhanush Haspula *et al.*

Corresponding author: Jürgen Wess, jurgenw@niddk.nih.gov

Sci. Adv. **11**, eadu1670 (2025)
DOI: 10.1126/sciadv.adu1670

This PDF file includes:

Figs. S1 to S21
Tables S1 and S2

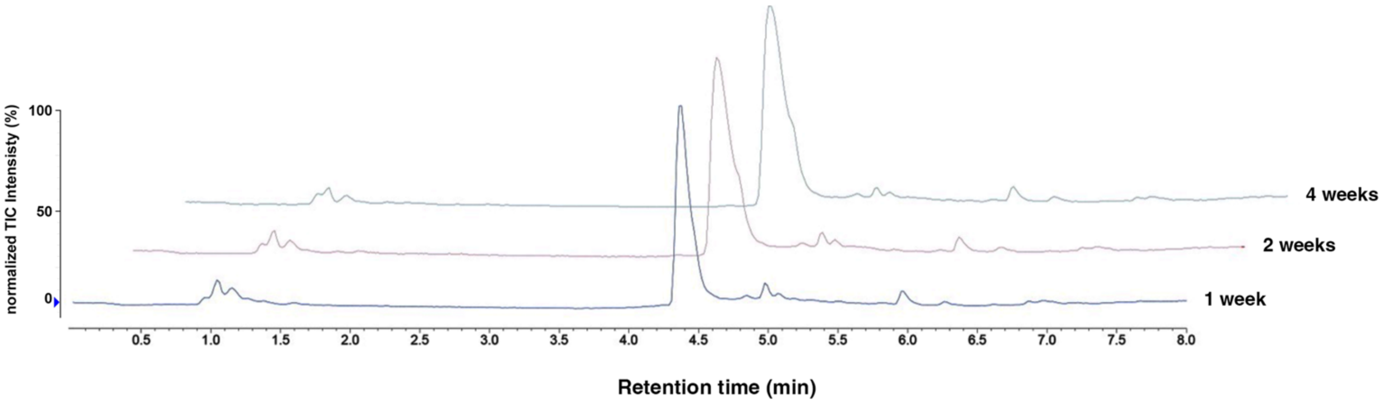

Fig. S1. DCZ is stable in aqueous solution. DCZ was dissolved in water at a concentration of 10 µg/ml. DCZ stability was tested via LS-MS after storing the solution for 1, 2, and 4 weeks at room temperature (23 ºC) (for details, see Materials and Methods). The data shown are representative of two independent experiments. TIC, Total Ion Chromatogram.

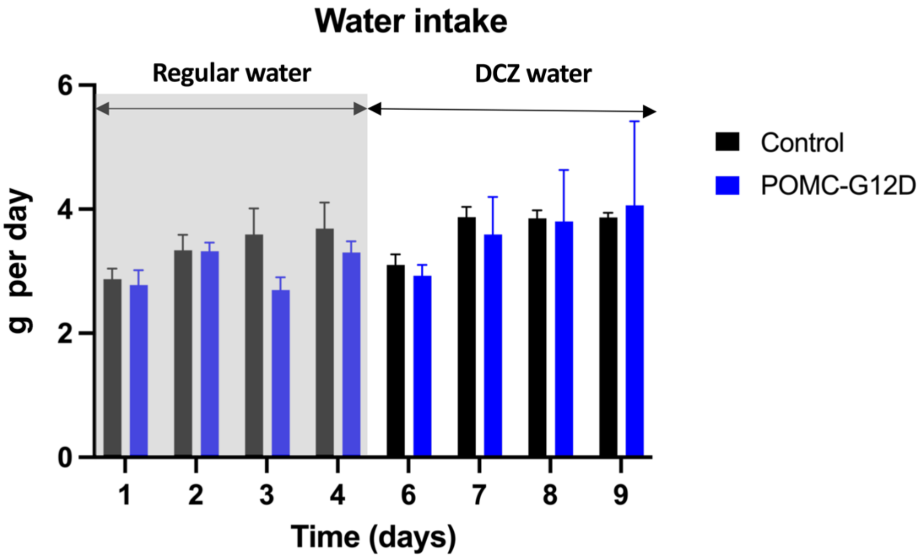

Fig. S2. DCZ water consumption does not differ between POMC-G12D and control mice. POMC-G12D mice and control littermates (age: 20 weeks) were maintained on regular drinking water for 4 days. Regular drinking water was then replaced with DCZ water for the following 4 days. Water consumption was measured daily. Data are presented as means ± SEM (n=5/group).

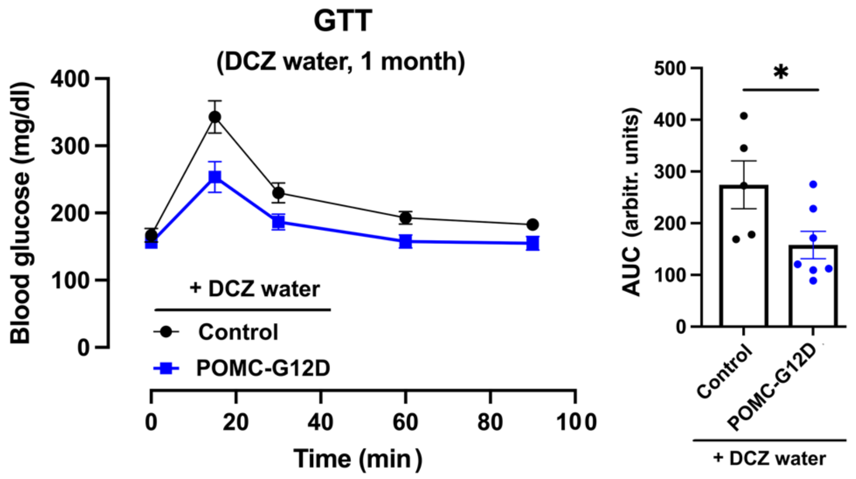

Fig. S3. DCZ retains biological activity in POMC-G12D mice consuming DCZ water for 1 month. POMC-G12D mice and control littermates lacking G12D (age: 26 weeks) were maintained on DCZ water for 1 month (POMC-G12D, n=8; control, n=5). After this time, all mice were subjected to a GTT (1.5 g glucose/kg, i.p.). Data are presented as means ± SEM. *P<0.05 (unpaired t-test). AUC, area under the curve.

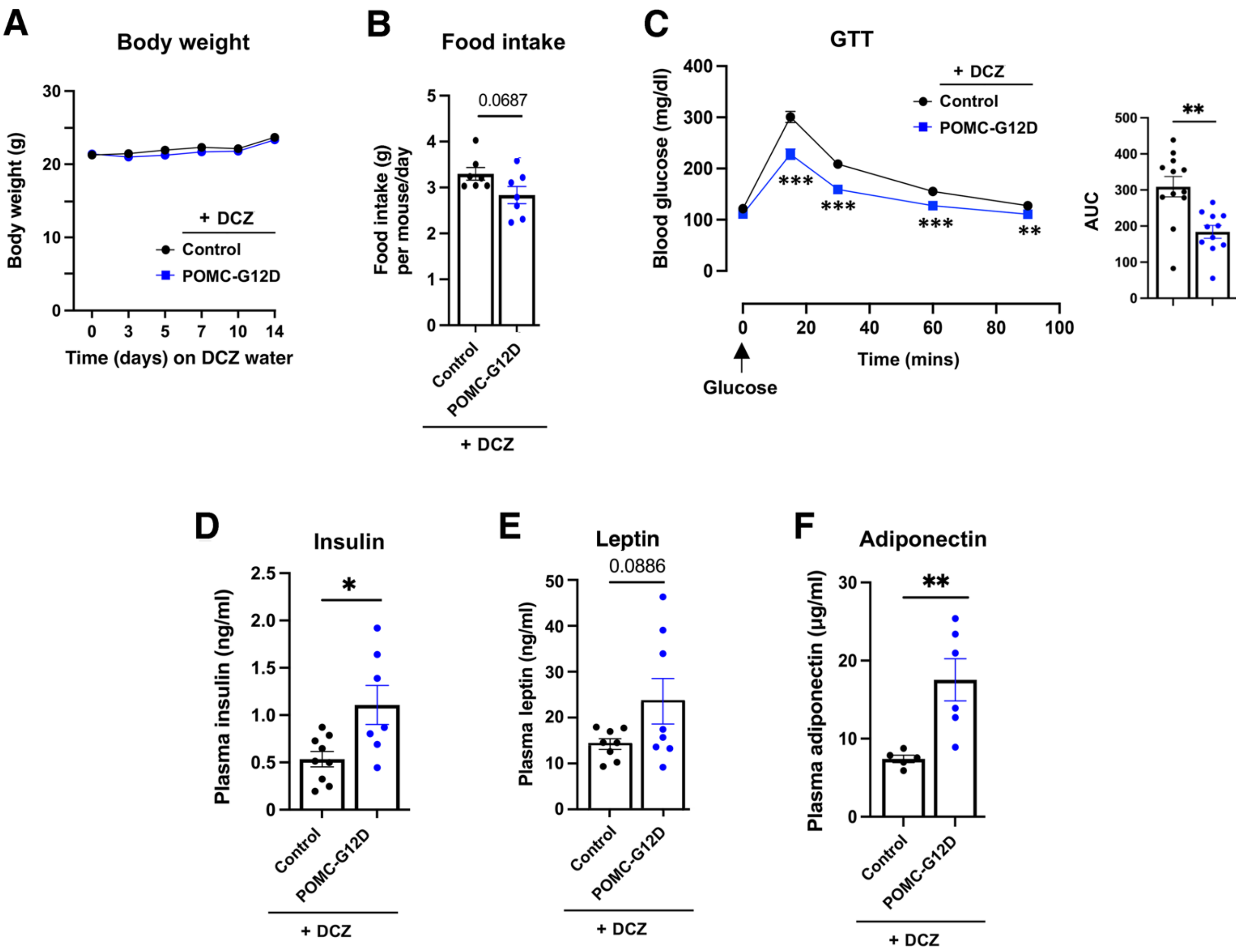

Fig. S4. Chronic stimulation of G12D signaling in female POMC-G12D mice causes similar metabolic changes as in their male counterparts. All studies were carried out with female mice consuming regular chow. **(A, B)** Body weight (A) and food intake (B) of POMC-G12D mice and control littermates maintained on DCZ drinking water (10 µg/ml) for up to two weeks. Panel (B) shows average food intake/day/mouse after 7 days of the DCZ treatment. **(C)** GTT (1.5 g glucose /kg, i.p.) carried out with POMC-G12D and control mice after consumption of DCZ water for 2 weeks. **(D-F)** Plasma insulin (D), leptin (E), and adiponectin (F) levels of freely fed POMC-G12D and control mice after treatment with DCZ water for 2 weeks. Experiments were performed with 8-11-week-old mice (n=6-12/group). Data are given as means ± SEM. *$P<0.05$,**$P<0.01$, ***$P<0.001$, as compared with the corresponding control group (two-way repeated measures ANOVA, followed by Šídák's multiple comparisons test (A, C); unpaired t-test (B, D-F)). Numbers above horizontal bars represent P values. ns, no statistically significant difference.

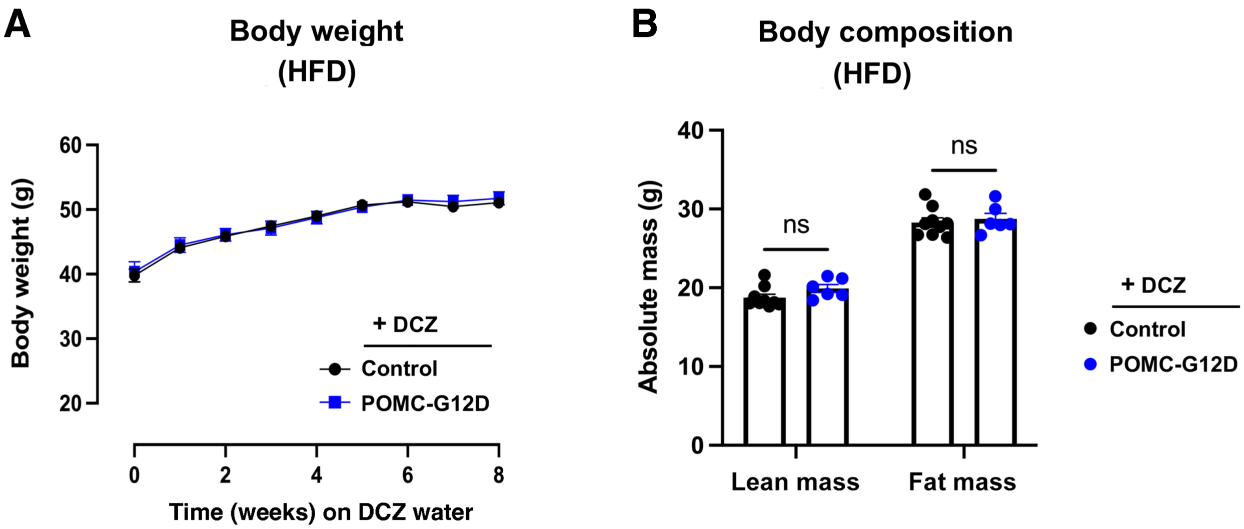

Fig. S5. POMC-G12D mice and control littermates consuming a high-fat diet (HFD) do not differ in body weight gain and body composition. All experiments were carried out with male mice consuming a HFD. (A) Body weight gain of POMC-G12D mice and control littermates maintained on a HFD. Mice consumed DCZ drinking water (10 μg/ml) during the HFD feeding period. HFD feeding was initiated when the mice were 20 weeks old. (B) Body composition data obtained after 8 weeks of HFD feeding and DCZ water consumption. Data are given as means ± SEM (n=6-9/group). Data were analyzed via two-way repeated measures ANOVA, followed by Šídák's multiple comparisons (A) or two-way ANOVA, followed by Šídák's multiple comparisons test (B), respectively. ns, no statistically significant difference.

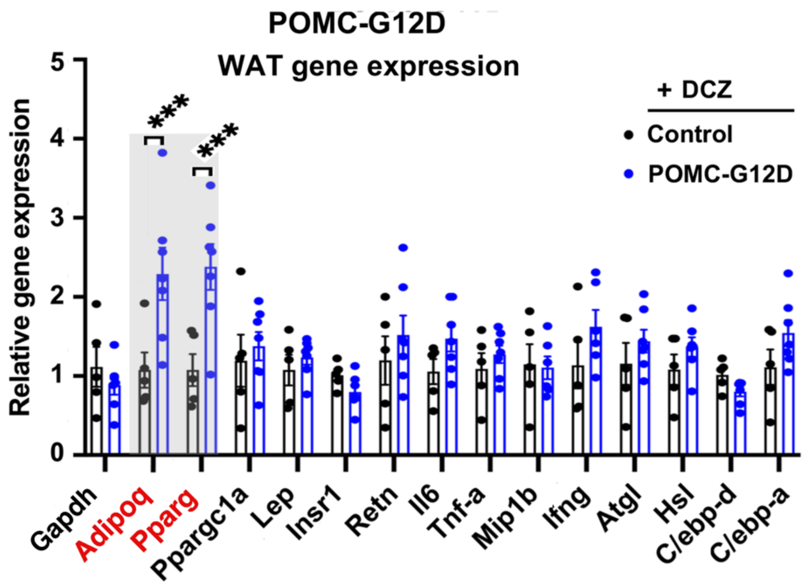

Fig. S6. **G12D-mediated changes in gene expression in white adipose tissue.** Gene expression was studied via q-RT-PCR using RNA prepared from eWAT of POMC-G12D mice and control littermate maintained on DCZ water for 4 weeks. Note that *Pparg* and *Adipoq* transcript levels are significantly increased in eWAT from POMC-G12D mice. For emphasis, these latter data are also shown in main Fig. 2I. Tissues were collected from mice (age: ~20 weeks) maintained on DCZ water for 4 weeks (n=4-7/group). Data are given as means ± SEM. ***P<0.001, as compared with the corresponding control group (two-way ANOVA, followed by Šídák's multiple comparisons test).

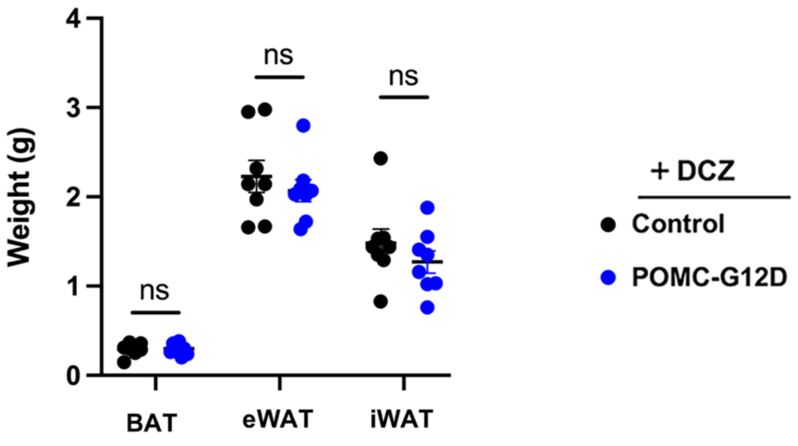

Fig. S7. **Weights of adipose tissue weights after chronic DCZ treatment of POMC-G12D mice**. All experiments were carried out with mice consuming regular chow.
Weights of adipose tissue depots of POMC-G12D mice and control littermates maintained on DCZ water for 4 weeks (n=6-9/group; ~20-week-old female mice). Data are given as means ± SEM. ns, no statistically significant difference (two-way ANOVA, followed by Šídák's multiple comparisons test).

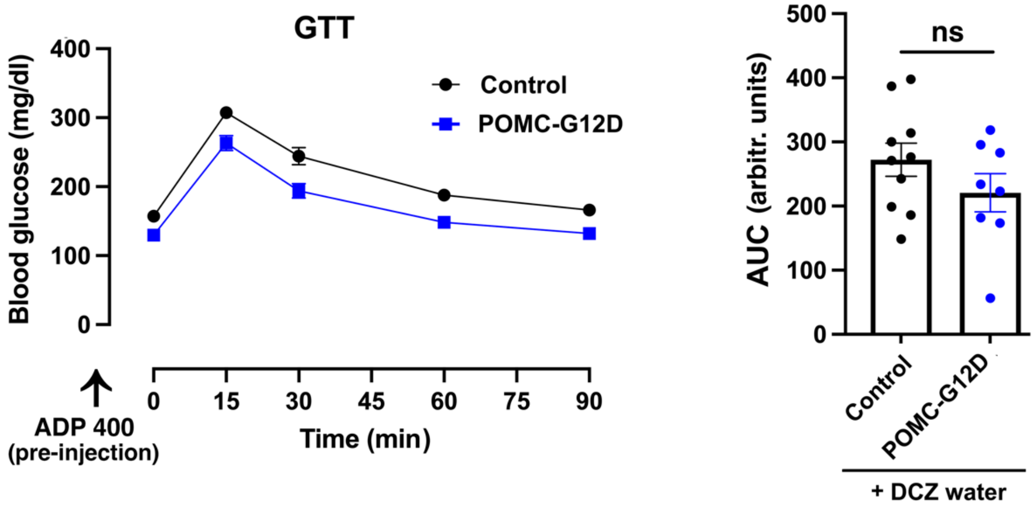

Fig. S8. DCZ-mediated improvement in glucose tolerance is absent after treatment of POMC-G12D mice with an adiponectin receptor antagonist. POMC-G12D mice and control littermates lacking G12D were subjected to an i.p. GTT (1.5 g glucose/kg, i.p.) following consumption of DCZ water for 2 weeks. Prior to administration of the glucose bolus, all mice received two doses of an adiponectin receptor antagonist (ADP 400; 1 mg/kg, s.c.; 1 and 7 hr prior to glucose) (mouse age: 12 weeks; n=8 or 9 per group). Data are presented as means ± SEM. AUC, area under the curve; ns, no statistically significant difference (unpaired t-test).

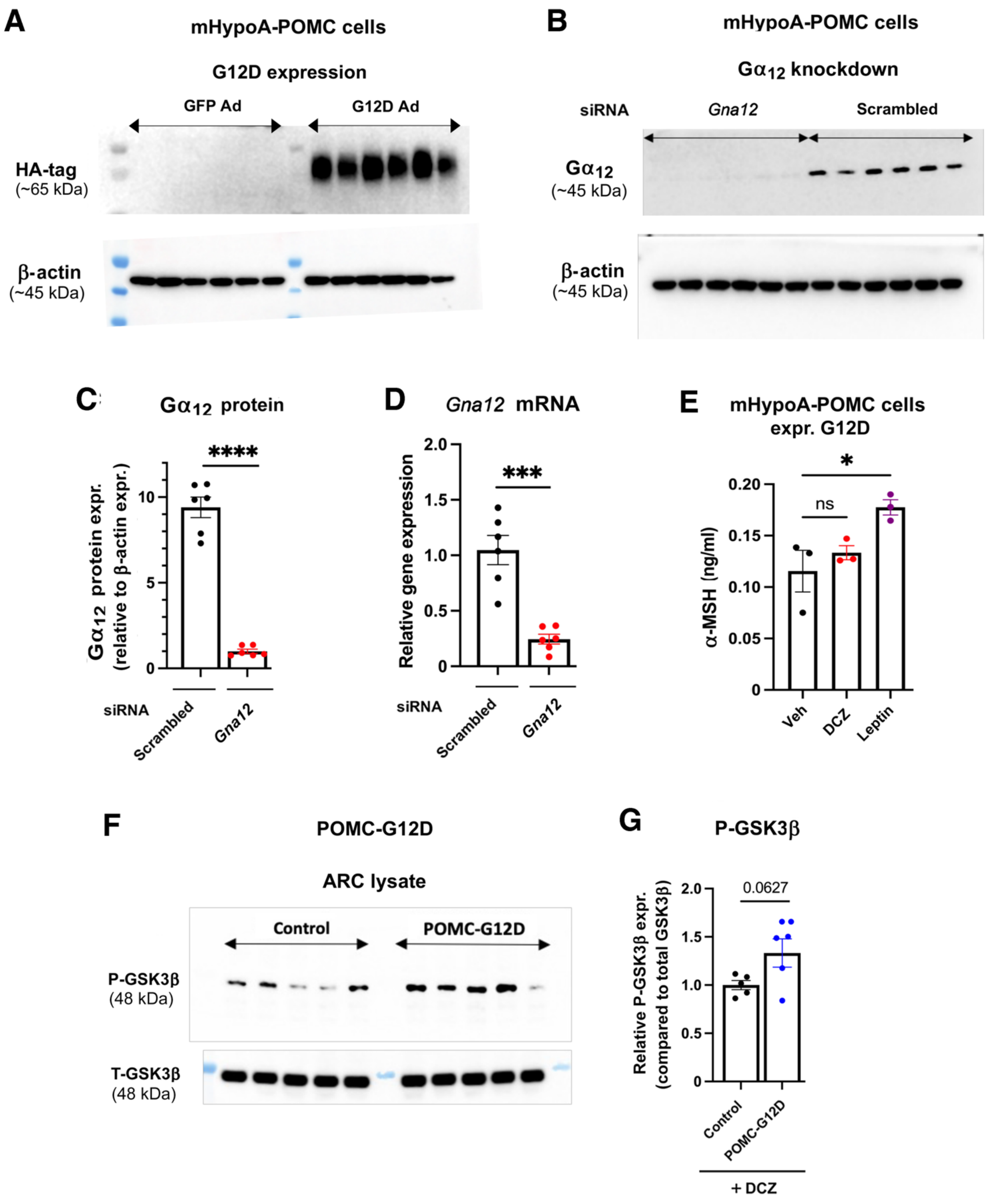

Fig. S9. **G12D-mediated effects in vitro and in vivo.** All in vivo and ex vivo experiments were carried out with male mice maintained on regular chow. **(A)** Immunoblot demonstrating the expression of the HA-tagged G12D receptor in mHypoA-POMC cells. Cells were treated with an

adenovirus coding for G12D (G12D Ad) or with an adenovirus encoding eGFP (GFP Ad; control cells). (**B**) Western blot demonstrating the efficient knockdown of $G\alpha_{12}$ expression after transfection of mHypoA-POMC cells with *Gna12* siRNA. (**C**) Quantification of the immunoblotting data show in (B). (**D**) qRT-PCR data showing the robust decrease in *Gna*12 expression following transfection of mHypoA-POMC cells with *Gna12* siRNA. (**E**) α-MSH secretion from mHypoA-POMC cells expressing G12D. Cells were treated with either DCZ (50 nM) or leptin (100 nM) for 30 min. Subsequently, α-MSH levels were measured in the medium. (**F**) Western blot demonstrating that activation of G12D in ARC POMC neurons stimulates GSK3β phosphorylation. ARC protein lysates were prepared from POMC-G12D mice and control littermates maintained on DCZ water (10 μg/ml) for 4 weeks (n=5). (**G**) Quantification of the immunoblotting data shown in (F). The number of n in (A-E) was 3-6. Results presented in (F, G) were generated using ARC lysates collected from 20-week-old mice maintained on DCZ water for 4 weeks (n=4-7/group). Data are given as means ± SEM. *P<0.05, ***P<0.001, ****P<0.0001, as compared with the corresponding control group (two-way ANOVA, followed by Šídák's multiple comparisons test (E); unpaired t-test (C, D, G)). Numbers above horizontal bars represent P values. ns, no statistically significant difference.

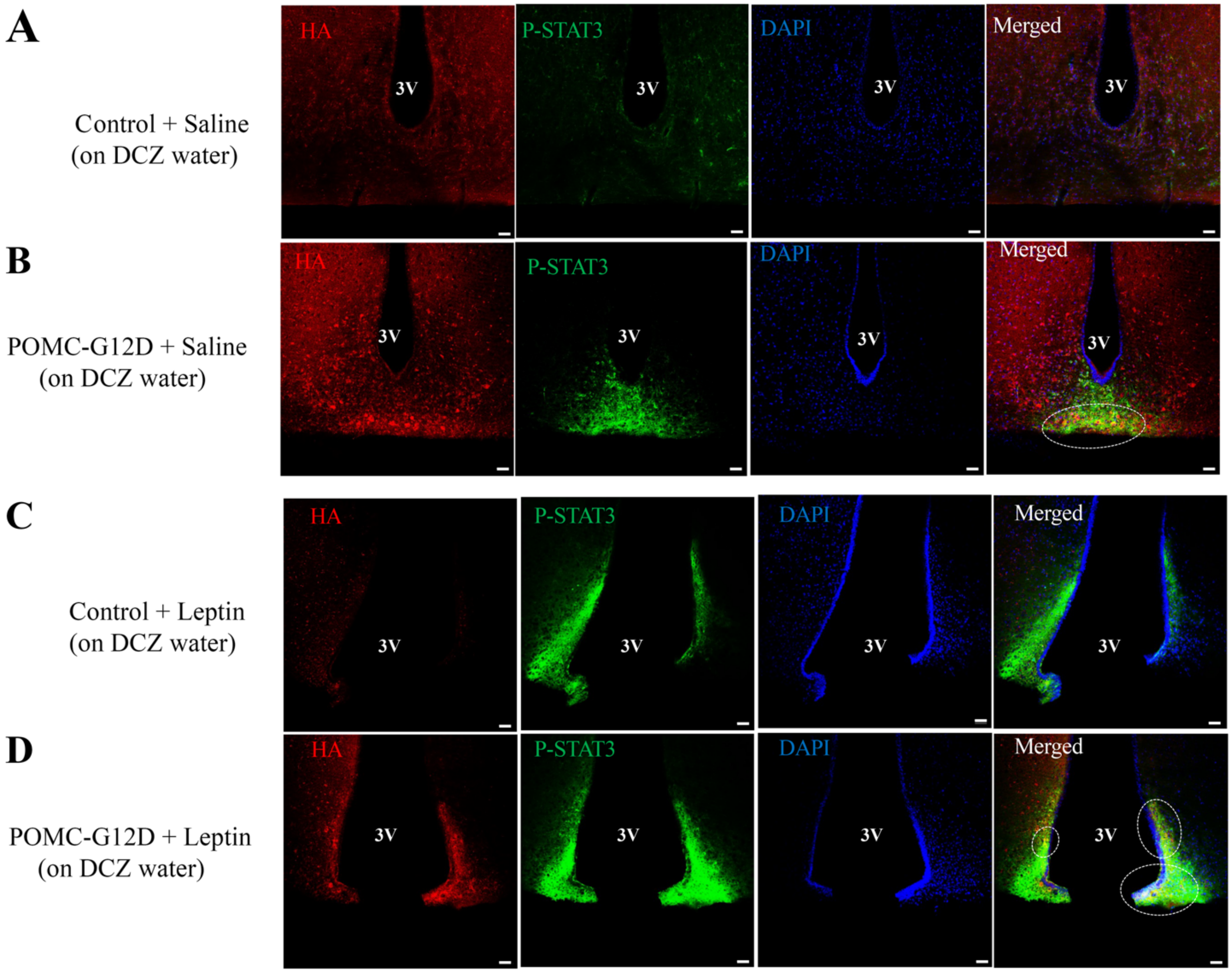

Fig. S10. G12D- and leptin-induced formation of p-STAT3 in ARC POMC neurons. **(A-D)** Representative images displaying the expression of p-STAT3 in the ARC of control mice lacking G12D (A, C) and POMC-G12D mice. All mice were maintained on DCZ water (10 mg/l) for 3 consecutive days. After an overnight fast (16 hr), mice were injected with either saline (A, B) or leptin (5 mg/kg, i.p.) (C, D). Brain tissues were collected 1 hr later. G12D-expressing neurons were visualized by using an anti-HA antibody (red) and co-stained for p-STAT3 (green) and DAPI (blue). Dotted ovals indicate colocalization of p-STAT3 with POMC/G12D+ neurons. Note that the faint red staining in the control slices (A, C; left panels) treated with the anti-HA antibody represents background staining. 12-15 sections from 2 or 3 different mice were analyzed. Scale bars, 100 µm. 3V, third ventricle.

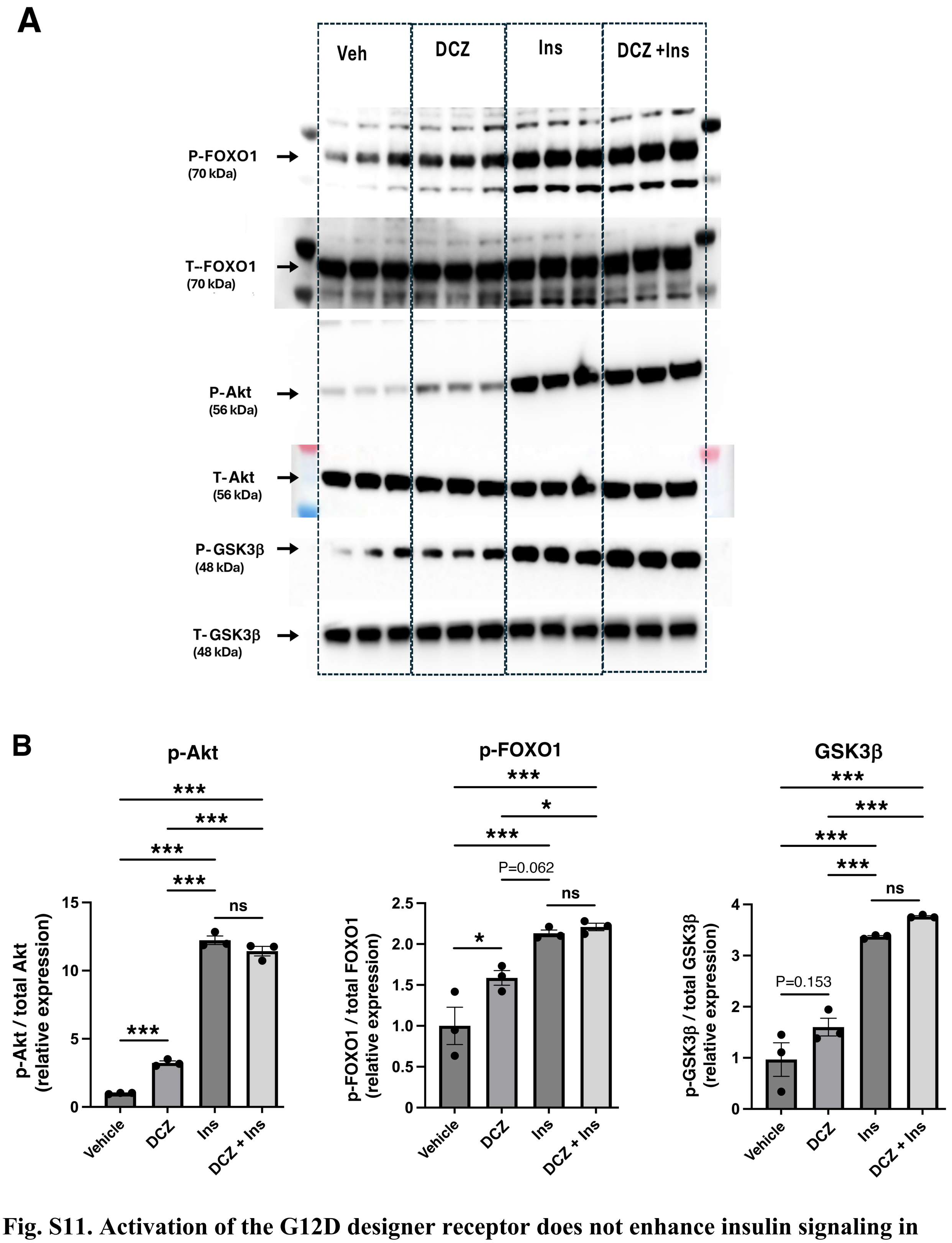

Fig. S11. Activation of the G12D designer receptor does not enhance insulin signaling in vitro. (A, B) G12D-expressing mHypoA-POMC cells were incubated for 15 min with either vehicle (Veh), DCZ (50 nM), insulin (Ins, 100 nM), or a mixture of DCZ and Ins (50 and 100

nM, respectively). (A) Cell lysates were subjected to Western blotting experiments using antibodies against the indicated proteins. (B) Quantification of the immunoblotting data shown in (A). Data are presented as means ± SEM (n=3). *P<0.05, *** P<0.001, as compared with the corresponding control group (one-way ANOVA, followed by Tukey's multiple comparison test). ns, no statistically significant difference.

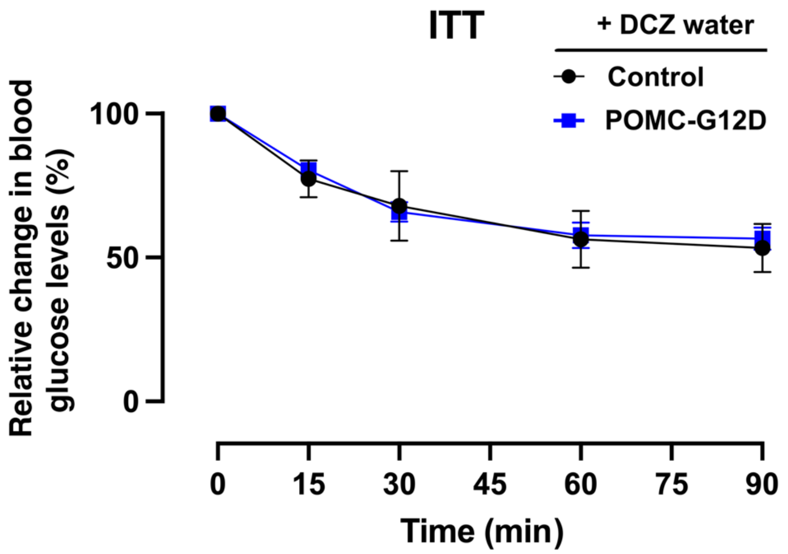

Fig. S12. POMC-G12D mice consuming DCZ water show unchanged insulin tolerance. POMC-G12D mice and control littermates lacking G12D that had been maintained on DCZ water (10 μg/ml) for 2 weeks were subjected to an ITT (1 U insulin/kg, i.p.). The figure shows relative changes of blood glucose levels, as compared to baseline (100%) (mouse age: 8 weeks; control, n=5; POMC-G12D, n=8). Data are presented as means ± SEM.

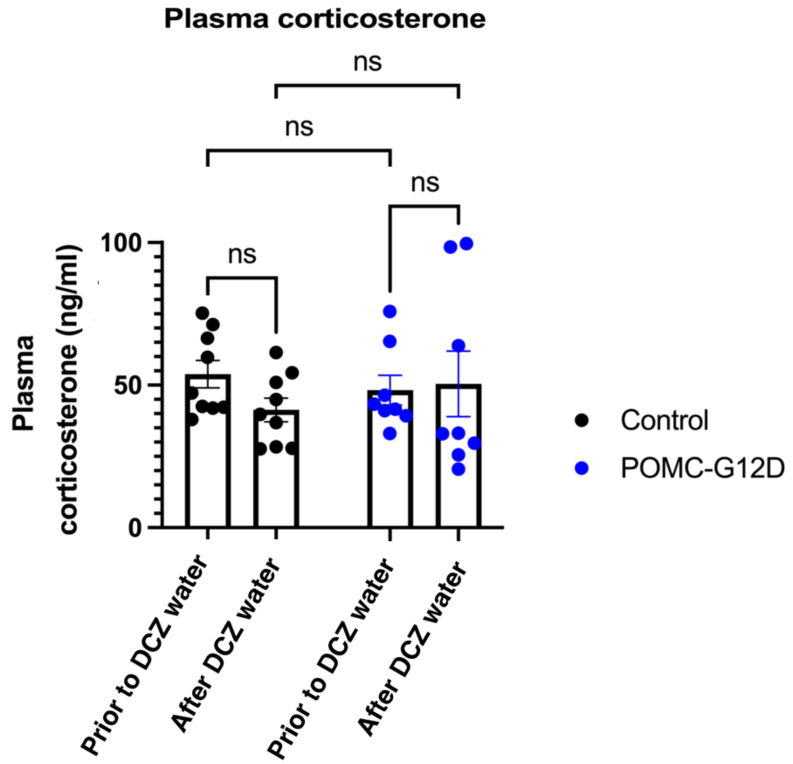

Fig. S13. **Chronic DCZ treatment of POMC-G12D mice does not affect plasma corticosterone levels.** Plasma corticosterone levels of POMC-G12D mice and control littermates maintained on DCZ water for 2 weeks. Plasma was obtained from 8-9-week-old male mice. Data are given as means ± SEM (n=8 or 9/group). ns, no statistically significant difference (two-way ANOVA, followed by Šídák's multiple comparisons test).

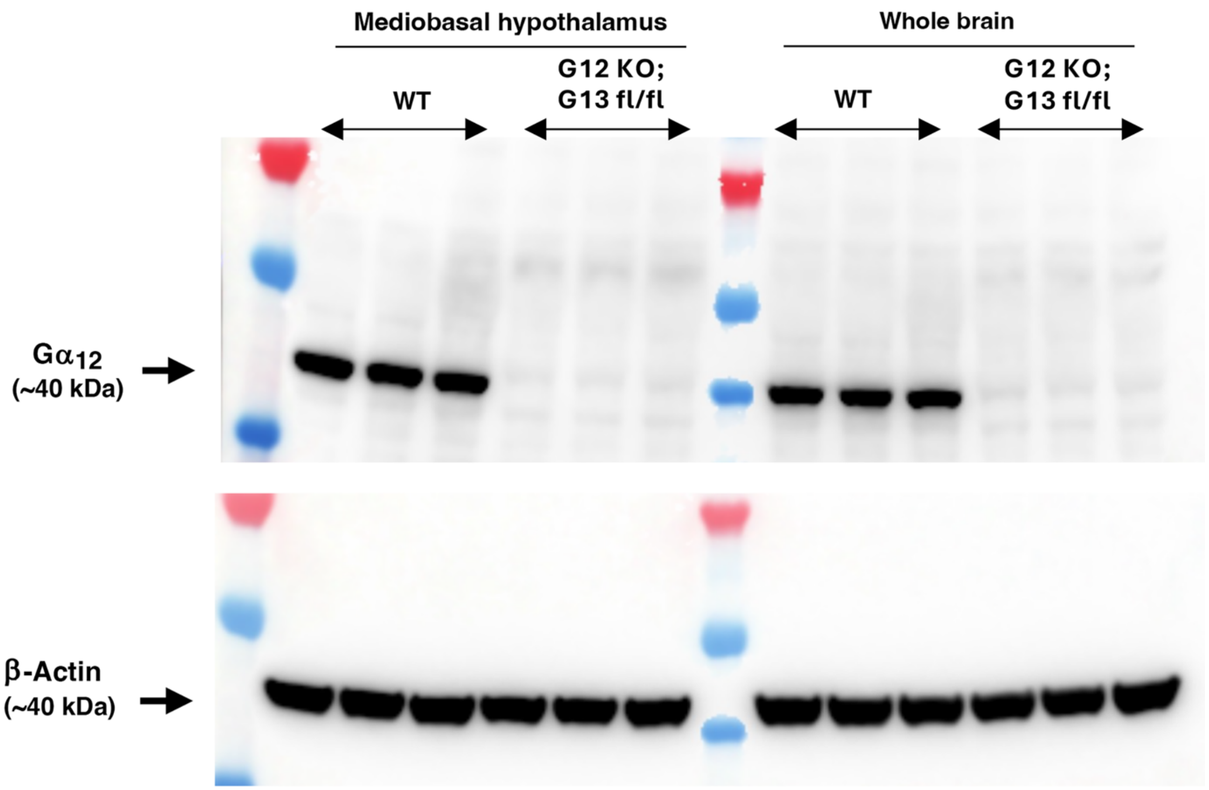

Fig. S14. Western blots demonstrating the absence of Gα$_{12}$ protein in tissues from G12 KO mice. Immunoblotting studies were carried out using lysates from the mediobasal hypothalamic and whole brain prepared from wildtype (WT) and G12 KO:G13 fl/fl mice (n=3/group). Protein lysates were subjected to Western blotting studies using the indicated antibodies.

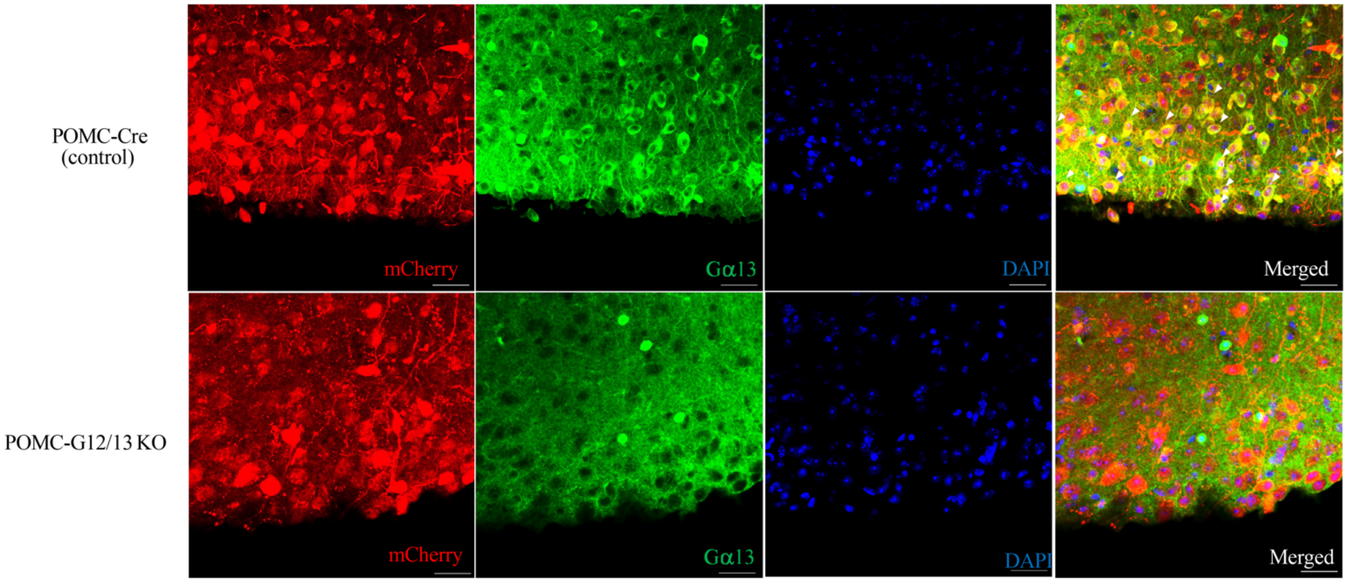

Fig. S15. Lack of Gα_{13} expression by ARC POMC neurons of POMC-G12/13 KO mice. The figures show representative images displaying the expression of Gα_{13} in POMC neurons of POMC-ARC-mCherry and POMC-G12/13 KO mice. In both groups of mice, ARC POMC neurons were visualized via mCherry fluorescence (red) (see Materials and Methods). Sections were stained for Gα_{13} (green) and DAPI (blue). White arrows indicate the expression of Gα_{13} by POMC neurons. 12-15 sections from two different mice were analyzed. Scale bars, 100 μm.

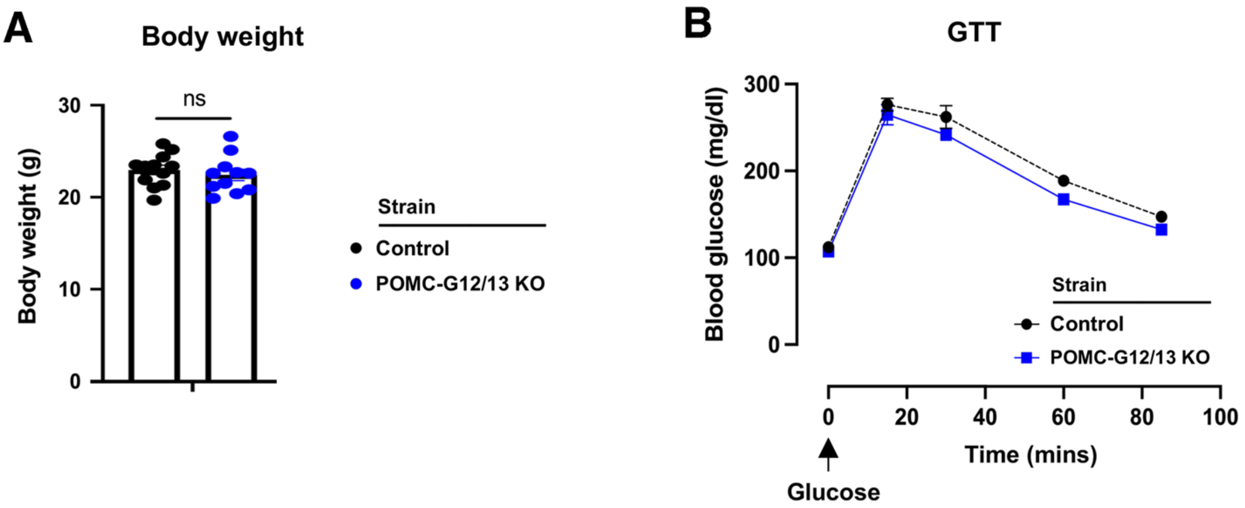

Fig. S16. POMC-G12/13 KO mice show similar body weight and glucose tolerance as control littermates. Experiments were carried out with male mice consuming regular chow (age: 12-14 weeks). **(A)** Body weight of mice deficient in $G\alpha_{12}$ and lacking $G\alpha_{13}$ selectively in POMC neurons (POMC-G12/13 KO mice). Littermates lacking only $G\alpha_{12}$ (*Gna12-/- Gna13 fl/fl* mice) served as control mice. (**B**) GTT (1.5 g glucose/kg, i.p.) carried out with POMC-G12/13 KO mice and control littermates. Data are presented as means ± SEM (n=11-13). ns, no statistically significant difference (unpaired t-test).

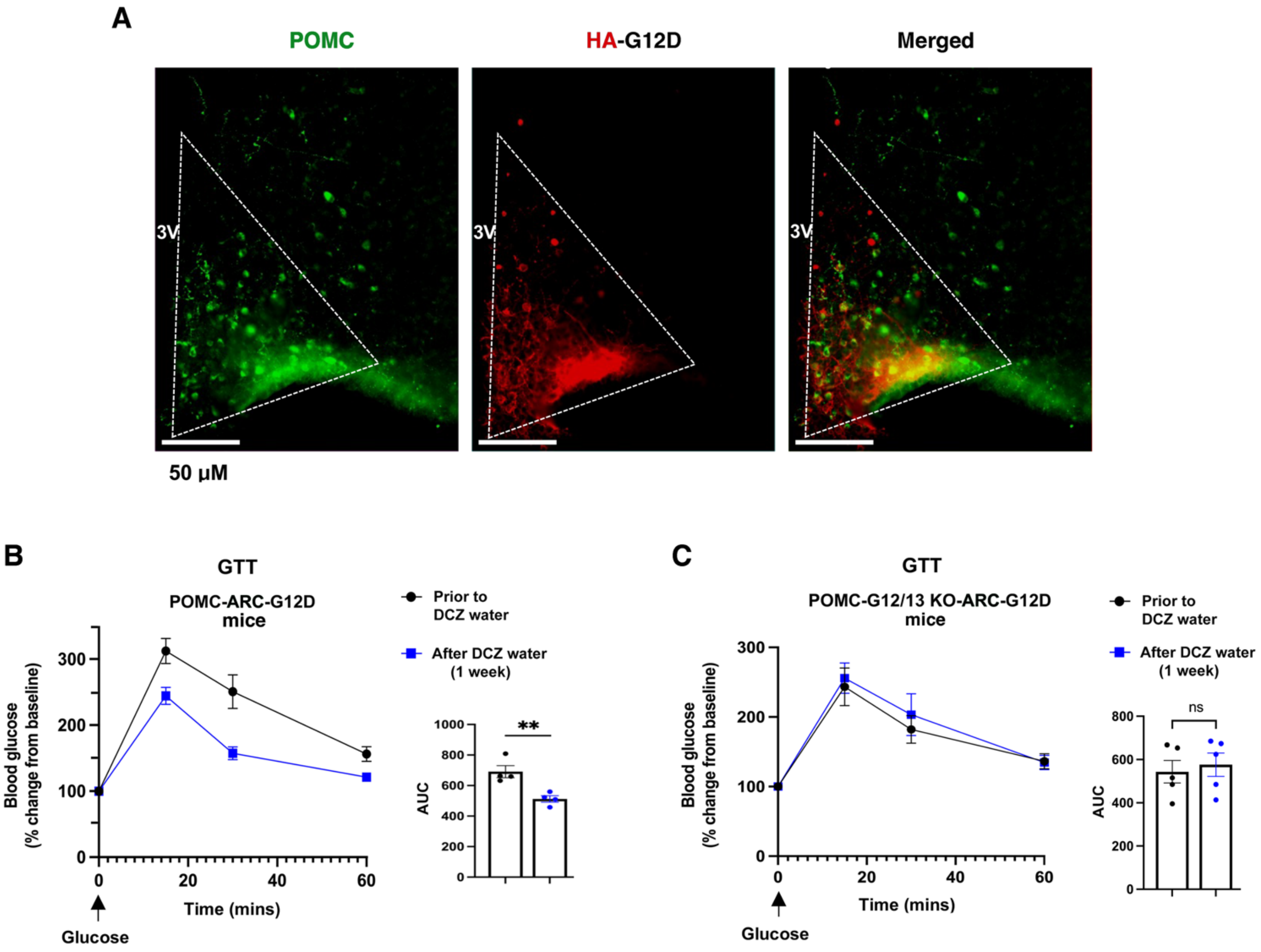

Fig. S17. G12D-mediated improvement in glucose tolerance is abolished in POMC-G12/13 KO mice. (**A**) Representative images demonstrating that microinjection of the AAV8.hSyn.DIO.HA-G12D-mCherry virus into the ARC of POMC-Cre mice results in the expression of the HA-G12D designer receptor in POMC neurons. The G12D receptor was visualized by using an antibody directed against the HA epitope tag that had been fused to the N-terminus of G12D (Fig. 1A). (**B, C**) GTT experiments (1.5 g glucose/kg, i.p.) were performed with the indicated mouse strains maintained on regular chow. Glucose tolerance was assessed prior to DCZ treatment and 1 week after consumption of DCZ (10 mg/ml) drinking water. Data are presented as % of basal blood glucose levels prior to glucose treatment. **(B)** POMC-ARC-G12D mice (mice expressing G12D selectively in ARC-POMC neurons). (C) POMC-G12/13 KO-ARC-G12D mice (mice expressing G12D selectively in ARC-POMC neurons of POMC-G12/13 KO mice). Experiments were carried out with 20-week-old female mice (n=4 or 5/group). Data are presented as means ± SEM. **P<0.01, as compared with the corresponding control group (unpaired t-test). ns, no statistically significant difference; 3V, third ventricle.

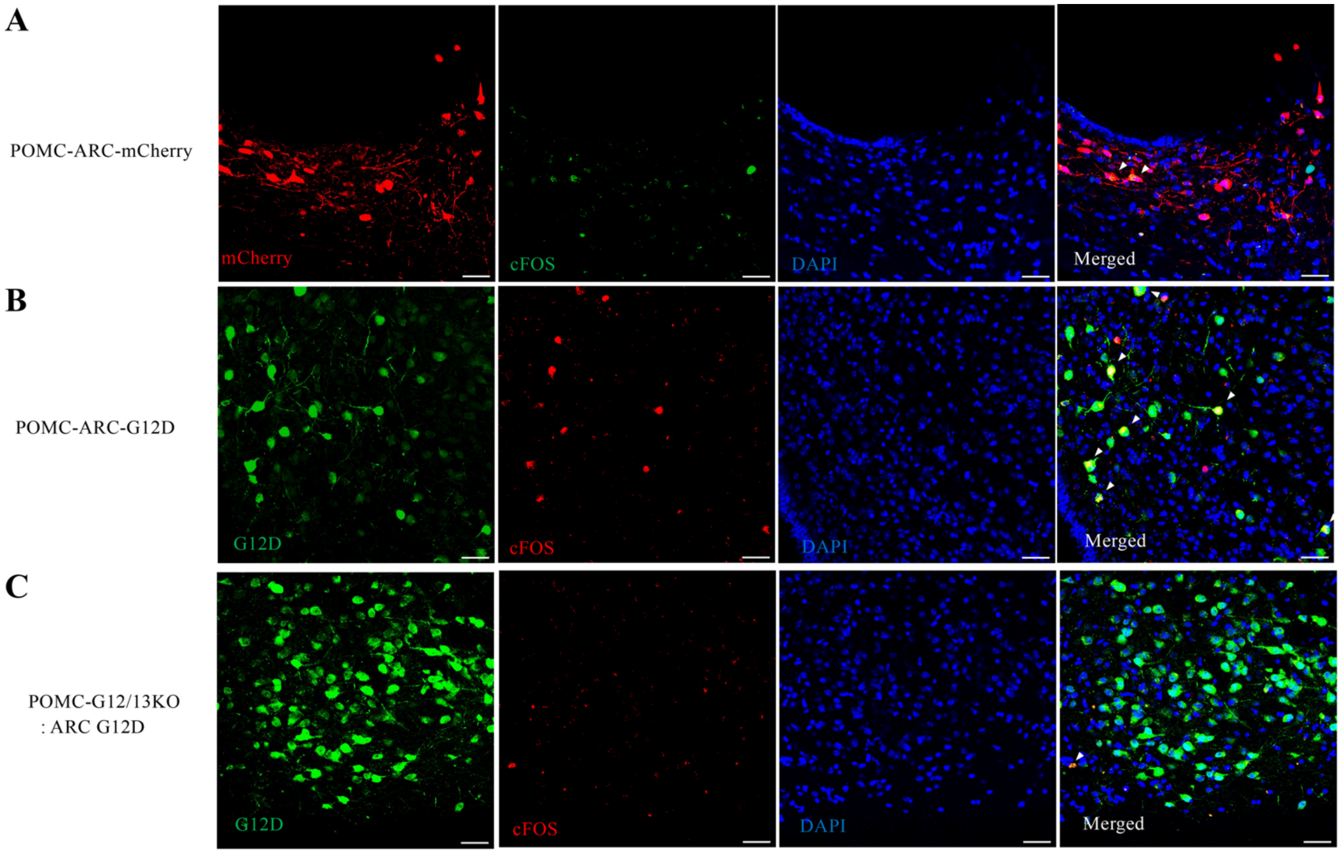

Fig. S18. G12D-mediated c-Fos activation in ARC POMC neurons is dependent on $G_{12/13}$ signaling. (A-C) Representative images showing c-Fos signals in the ARC of POMC-ARC-mCherry, POMC-ARC-G12D, and POMC-G12/13 KO ARC-G12D mice. The genotypes and generation of the different mouse strains are described under Materials and Methods. All mice were injected with DCZ (100 μg/kg, i.p.), and brains were collected 1 hr later. (A) POMC-ARC-mCherry mice. POMC neurons were visualized by mCherry fluorescence (red) and stained for c-Fos (green) and DAPI (blue) expression. (B, C) POMC-ARC-G12D and POMC-G12/13 KO ARC G12D mice. Sections were stained with a RFP-FITC antibody to visualize G12D expression in POMC neurons (green, note that the AAV used to express G12D in POMC neurons contained an mCherry reporter sequence). The sections were co-stained for c-Fos (red) and DAPI (blue). White arrows indicate colocalization of c-Fos with POMC/G12D+ neurons. 12-15 sections from 2 or 3 different mice were analyzed. Scale bars, 100 μm.

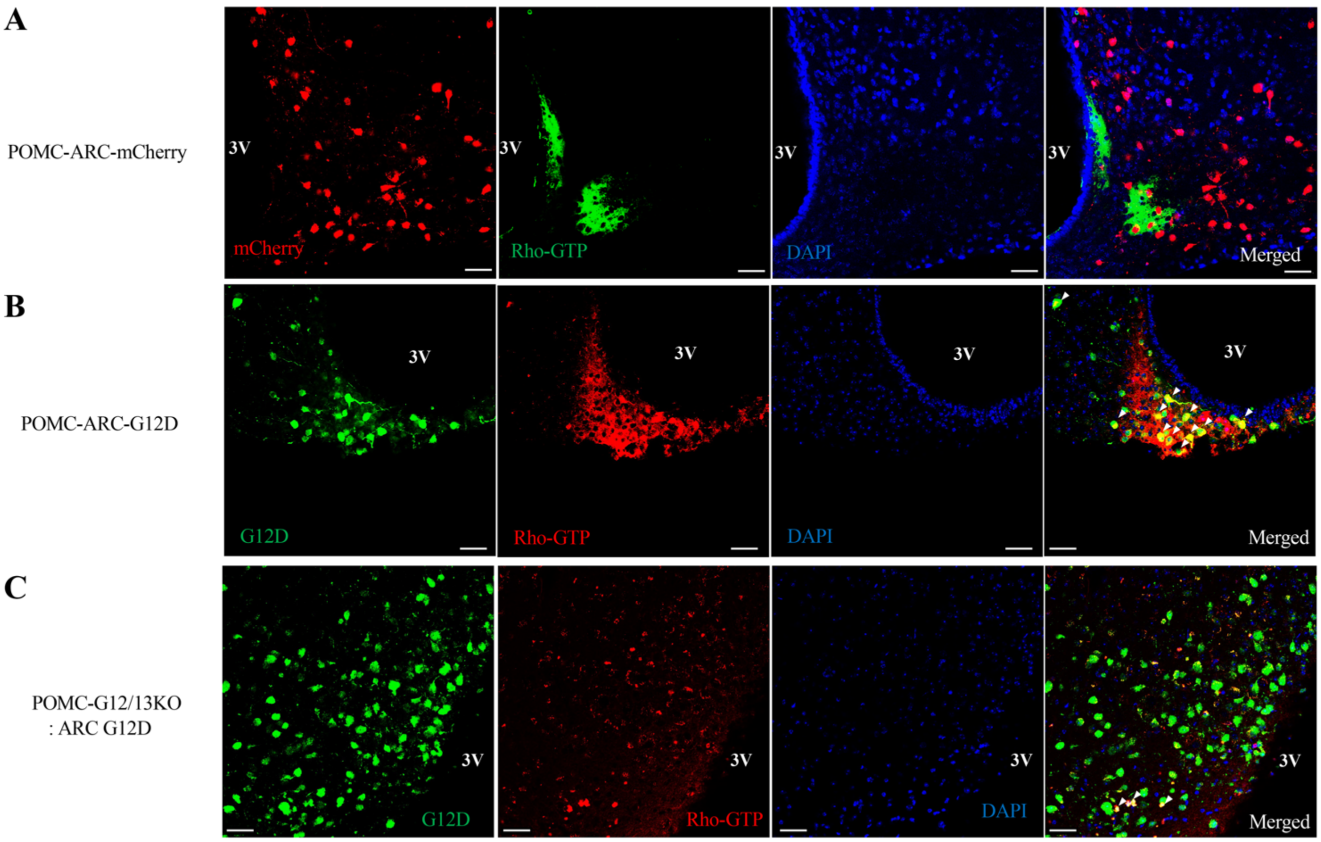

Fig. S19. G12D-mediated formation of RhoA-GTP in ARC POMC neurons is dependent on $G_{12/13}$ signaling. (A-C) Representative images displaying the expression of RhoA-GTP in the ARC of POMC-ARC-mCherry (A), POMC-ARC-G12D (B), and POMC-G12/13 KO:ARC-G12D mice (C) are shown. All mice were injected with DCZ (100 μg/kg, i.p.), and brain tissues were collected 1 hr later. The genotypes and generation of the different mouse strains are described under Materials and Methods. (A) POMC-ARC-mCherry mice. POMC neurons were visualized by mCherry fluorescence (red) and stained for RhoA-GTP (green) and DAPI (blue) expression. (B, C) POMC-ARC-G12D (and POMC-G12/13 KO:ARC G12D mice. Sections were stained with an RFP-FITC antibody to visualize G12D expression in POMC neurons (green; note that the AAV used to express G12D in POMC neurons contained an mCherry reporter sequence). RhoA-GTP and DAPI staining are shown in red and blue, respectively. White arrows indicate colocalization of RhoA-GTP with POMC neurons expressing G12D. 12-15 sections from 2 or 3 different mice were analyzed. Scale bars, 100 μm.

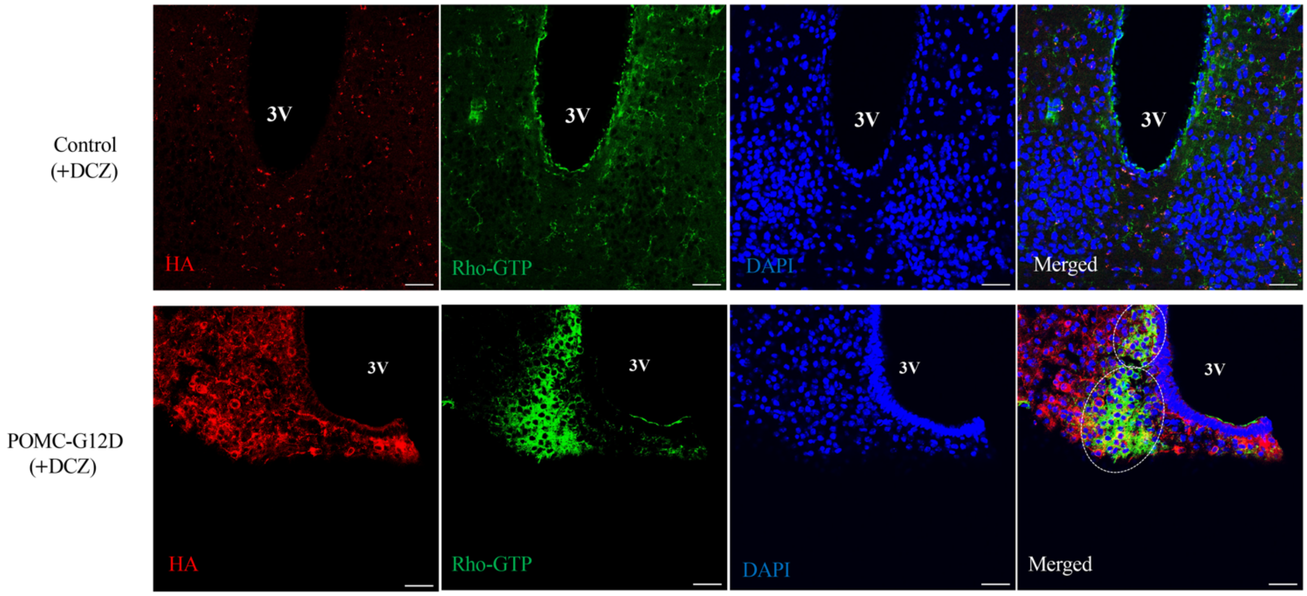

Fig. S20. G12D-mediated formation of RhoA-GTP in ARC POMC neurons after chronic G12D activation. The figure displays representative images showing the expression of RhoA-GTP in the ARC of control and POMC-G12D mice maintained on DCZ water (10 µg/ml) for 3 consecutive days. Hypothalamic slices were prepared after an overnight fast (16 hr). G12D expressing-neurons were visualized by using an anti-HA antibody (red) and co-stained for RhoA-GTP (green) and DAPI (blue). The dotted oval indicates colocalization of RhoA-GTP with POMC/G12D+ neurons. 12-15 sections from 2 or 3 different mice were analyzed. Scale bars, 100 µm. 3V, third ventricle.

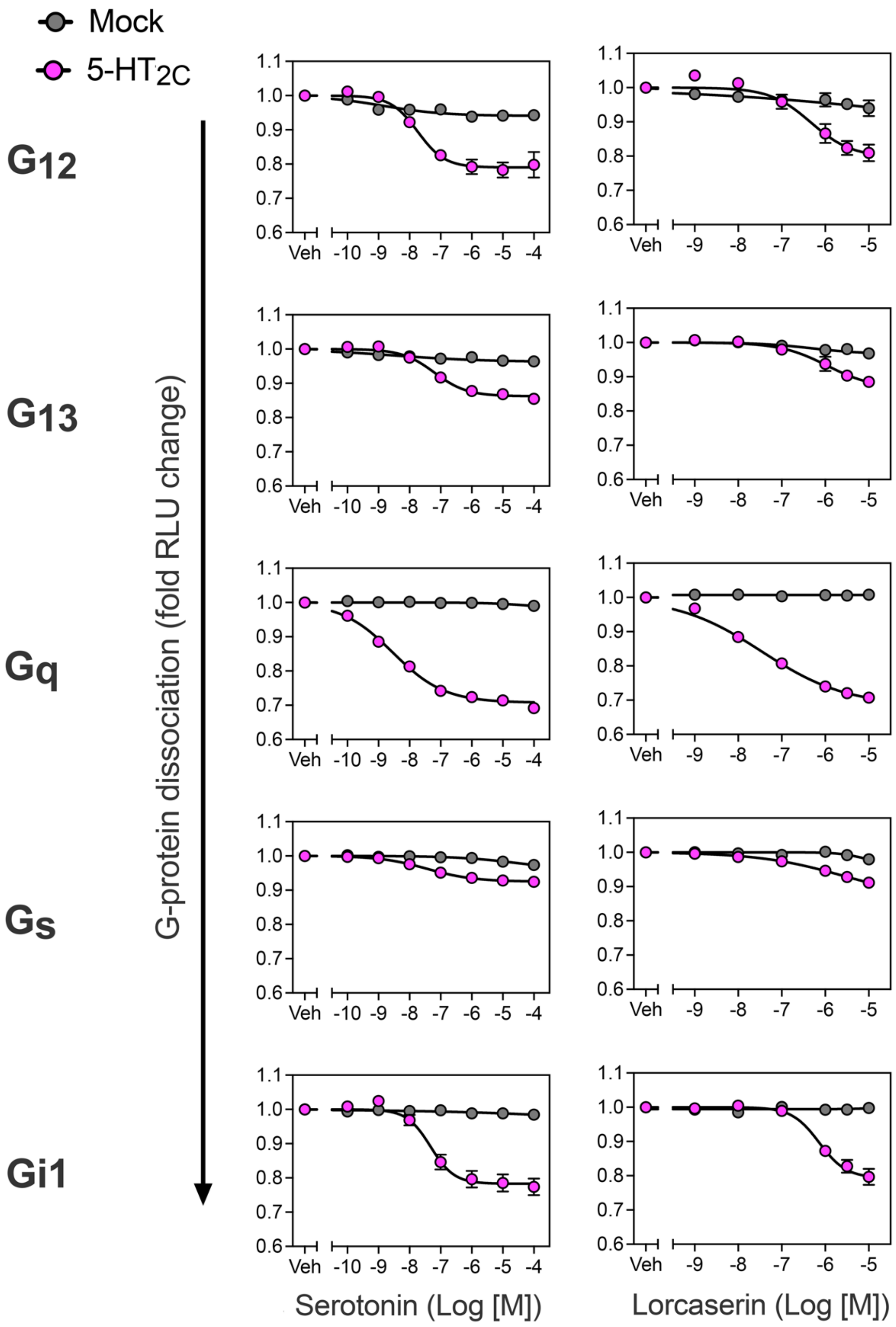

Fig. S21. G protein-coupling profiles after activation of 5-HT$_{2C}$ receptors by lorcaserin and serotonin in vitro. HEK293A cells expressing the 5-HT$_{2C}$ receptor (5-HT$_{2C}$) were treated with increasing concentrations of lorcaserin or serotonin. Mock-transfected HEK293 cells (mock)

served as control cells. G protein-coupling profiles were measured using NanoBiT-G protein dissociation assays, as described under Materials and Methods. Data represent means ± SEM from 3 to 4 independent experiments, each performed in duplicate. Veh, vehicle; RLU, Relative Light Units.

Table S1. Antibodies, drugs, reagents, kits, and mouse strains used in this study

Reagents	Source	Cat. # (identifier)
**Antibodies**		
Alexa Fluor 488	Cell Signaling	4412S
Alexa Fluor 594	Cell Signaling	8889S
AKT Rb Ab	Cell Signaling	9272S
p-AKT Rb mAb (T308)	Cell Signaling	4056S
β-actin mouse mAb	Santa Cruz	SC-47778
c-Fos guinea pig mAb	Synaptic Systems	226 308
ERK1/2 Rb Ab	Cell Signaling	9102
p-ERK1/2 Rb Ab	Cell Signaling	9101
FoxO1 mAb	Cell Signaling	2880S
p-FoxO1 Rb Ab	Cell Signaling	9461S
GSK3β Rb mAb	Cell Signaling	9315S
p-GSK3β Rb Ab	Cell Signaling	9336S
$G\alpha_{12}$ mouse mAb	Santa Cruz	SC-515445
$G\alpha_{13}$ mouse mAb	Proteintech	67188-1-Ig
HA-tag Rb mAb	Cell Signaling	3724S
HA-tag mouse mAb	Cell Signaling	2367
HRP-linked secondary Ab (Rb)	Cell Signaling	7074
HRP-linked secondary Ab (mouse)	Cell Signaling	7076
JAK2 Rb mAb	Cell Signaling	3230S
p-JAK2 Rb Ab	Cell Signaling	3771S
POMC Rb Ab	Phoenix Pharmaceuticals	H-029-30
RFP-FITC Rb Ab	Abcam	ab34764
RhoA mouse mAb	Cytoskeleton	ARH05
RhoA-GTP mouse mAb	NewEast Biosciences	26904
STAT3 Rb mAb	Cell Signaling	4904S
p-STAT3 Rb mAb	Cell Signaling	9145S
p-STAT3 mouse mAb	Cell Signaling	4113
**Chemicals, peptides, reagents**		
Agarose	Thermo Fisher Scientific	16500-500
ADP 400	Ambiopharm	
Bovine serum albumin	GoldBio	A-420-250
Clozapine N-oxide (CNO)	Advanced Molecular Technologies	AMTA056-CO16

Deschloroclozapine dihydrochloride (DCZ)	Hello Bio	HB9126
DMEM - high glucose	Sigma-Aldrich	D5796
EDTA	Sigma-Aldrich	E7889
Emerald AMP GT PCR Master Mix	Takara	RR310
Fetal bovine serum	Sigma-Aldrich	F4135
Insulin (Humulin R)	Eli Lilly	00002821501
Isoflurane	Baxter Healthcare Corporation	10019-360-40
Leptin	R&D Systems	498-OB-05M
Lipofectamine RNAimax	Thermo Fisher Scientific	13778075
Lorcaserin hydrochloride	AbMole	M2821
Meloxicam SR	ZooPharm	N/A
N-methylatropine bromide	Sigma-Aldrich	M1300000
NuPAGE 4-12% Bis-Tris protein gel	Invitrogen	NP0336BOX
NUPAGE MOPS SDS running buffer (20x)	Invitrogen	NP0001
NuPAGE 3-8% Tris-Acetate protein gel	Invitrogen	EA03785BOX
Nitrocellulose transfer packs	BioRad	1704158
NuPAGE LDS sample buffer (4X)	Invitrogen	NP0007
Opti-MEM	Thermo Fisher Scientific	31985070
(±)-Propranolol hydrochloride	Sigma-Aldrich	P0884
Penicillin-Streptomycin	Thermo Fisher Scientific	15140122
PhosStop	Sigma-Aldrich	4906845001
Protease Inhibitor Cocktail	Sigma-Aldrich	11836170001
PBS	KD Medical	RGF-3190
RIPA lysis and extraction buffer	Thermo Fisher Scientific	89900
SuperSignal West Dura Extended Duration Substrate	Themo Scientific	34076
SuperFemto ECL Chemiluminescence Kit	Vazyme	E423-02
iTaq™ Universal SYBR® Green Supermix	BioRad	1725121
Sodium metabisulfite	Sigma-Aldrich	161519
10x TBS	KD Medical	RGF-3385
Trypsin-EDTA solution	Sigma-Aldrich	T4049
Tween 20	Sigma-Aldrich	P1379
Vectashield	VectorLabs	H-1000-10

Y27632	Tocris	1254
**Commercial assays/kits**		
Adiponectin/Acrp30 ELISA	R&D Systems	MRP300
BCA protein assay kit	Thermo Scientific	23227
2-CAT ELISA	Rocky Mountain Diagnostics	BA E-6500R
Direct-zol RNA microprep	Zymo Research	R2063-A
Ultra-Sensitive Mouse Insulin ELISA Kit	Crystal Chem	90082
Leptin Quantikine ELISA Kit	R&D Systems	MOB00B
MSH, alpha (human, rat, mouse) - EIA Kit	Phoenix Pharmaceuticals	EK-043-01
Pancreatic Polypeptide (PP) ELISA Kit	Cloud-Clone Corp	CEB265Mu
Rho Activation Assay Biochem Kit	Cytoskeleton	BK036
ZymoScript RT PreMix Kit	Zymo Research	R3012-1-1
Mouse Corticosterone ELISA Kit	Crystal Chem	80556
**Cell lines**		
mHypoA-POMC/GFP-2	Cedarlane	CLU501
HEK293A	Thermo Fisher Scientific	R70507
**Experimental models: Animals**		
Gna12-/- Gna13 fl/fl mice	Provided by Dr. Stefan Offermanns (Germany)	N/A
POMC-Cre mice	The Jackson Laboratory	005965
Rosa26-LSL-G12D-IRES-GFP mice (short: LSL-G12D)	Generated in the lab of Dr. Asuka Inoue (Japan)	N/A
Wildtype C57BL/6 mice	Taconic	B6NTac
**Oligonucleotides**		
ON-TARGETplus Mouse *Gna12* (14673)	Horizon Discovery	L-043467-00-0005
ON-TARGETplus Non-targeting pool	Horizon Discovery	D-001810-10-05
Primers for genotyping and qRT-PCR	A complete list is provided in Table S2.	

**Viruses**		
AAV8.hSyn.DIO.HA-G12D-mCherry	Penn Vector Core	N/A
AAV8.hSyn.DIO.mCherry	Addgene	50459-AAV8
Ad-CMV-HA-G12D	Vector Biolabs	N/A
Ad-CMV-eGFP	Vector Biolabs	N/A
**Software and algorithms**		
Prism 7 (Version 10)	Graph Pad	
ImageJ (v1.54f)	NIH	
CorelDraw X8	Corel Corp	

Table S2. PCR primers used in the present study

**Gene**	**Species**	**Primer sequence (Forward)**	**Primer sequence (Reverse)**
Pomc	Mouse	5- CCCGCCCAAGGACAAGCGTT-3	5-CTGGCCCTTCTTGTGCGCGT-3
Gna12	Mouse	5-TCCATCATCCTCTTCCTCAAC-3	5-TCCTTCACAGCATGAAACAC-3
Th	Mouse	5-CGGGCTTCTCTGACCAGGCG-3'	5-TGGGGAATTGGCTCACCCTGCT-3
Gapdh	Mouse	5'-CATCACTGCCACCCAGAAGACTG-3'	5'-ATGCCAGTGAGCTTCCCGTTCAG-3'
Ppargc1a	Mouse	5'-TGACAAATGCTCTTCGCTTT-3'	5'-CAAACCCTGCCATTGTTAAG-3'
Lep	Mouse	5'-CAAGCAGTGCCTATCCAGA-3'	5-AAGCCCAGGAATGAAGTCCA-3'
Insr1	Mouse	5'-ATGGGCTTCGGGAGAGGAT-3	5'-GGATGTCCATACCAGGGCAC-3'
Retn	Mouse	5'-CTGTCCAGTCTATCCTTGCACAC-3'	5'-CAGAAGGCACAGCAGTCTTGA-3'
Il6	Mouse	5'-TAGTCCTTCCTACCCCAATTTCC-3'	5'-TTGGTCCTTAGCCACTCCTTC-3'
Tnfa	Mouse	5'-CCCTCACACTCAGATCATCTTCT-3'	5'-GCTACGACGTGGGCTACAG- 3'
Mip1b	Mouse	5'-AACAACATGAAGCTCTGCGT-3'	5'-AGAAACAGCAGGAAGTGGGA-3'
Ifng	Mouse	5'-CGGCACAGTCATTGAAAGCCTA-3'	5'-GTTGCTGATGGCCTGATTGTC-3'
Atgl	Mouse	5'-GGATGGCGGCATTTCAGACA-3'	5'-CAAAGGGTTGGGTTGGTTCAG-3'
Hsl	Mouse	5'-CCAGCCTGAGGGCTTACTG-3'	5'-CTCCATTGACTGTGACATCTCG-3'
C/ebp-d	Mouse	5'-CGACTTCAGCGCCTACATTGA-3'	5'-CTAGCGACAGACCCCACAC-3'
C/ebp-a	Mouse	5'-CAAGAACAGCAACGAGTACCG-3'	5'-GTCACTGGTCAACTCCAGCAC-3'
β-actin	Mouse	Qiagen, Cat # is QT01136772	

Gene	Species	Source	Unique assay ID
Agrp	Mouse	BioRad	qMmuCED0004115
Npy	Mouse	BioRad	qMmuCID0006819
Adipoq	Mouse	BioRad	qMmuCED0045486
Pparg	Mouse	BioRad	qMmuCID0018821
